# Disease Progression Modeling and Stratification for detecting sub-trajectories in the natural history of pathologies: Application to Alzheimer’s disease trajectory modeling

**DOI:** 10.1162/IMAG.a.954

**Published:** 2025-11-06

**Authors:** Alessandro Viani, Anna Custo, Emile d’Angremont, Valentina Garibotto, Giovanni B. Frisoni, Boris A. Gutman, Marco Lorenzi

**Affiliations:** Epione Research Team, Inria Center of Université Côte d’Azur, Sophia Antipolis, France; Division of Nuclear Medicine and Molecular Imaging, Geneva University Hospitals (HUG), Geneva, Switzerland; Department of Anatomy and Neurosciences, Amsterdam University Medical Center, Amsterdam, The Netherlands; Département de Readaptation et Gériatrie, University of Geneva, Geneva, Switzerland; Department of Biomedical Engineering, Illinois Institute of Technology, Chicago, IL, United States

**Keywords:** disease progression modeling, progression subtyping, Alzheimer’s disease

## Abstract

Quantifying the progression of degenerative diseases remains crucial for early diagnosis, prevention, and treatment. However, accurately modeling disease biomarker evolution is hindered by substantial variability in disease trajectories among individuals, driven by demographic, genetic, and lifestyle factors. This variability gives rise to heterogeneous phenotypic manifestations, underscoring the need for stratification based on underlying disease subtypes. Recent advances have shown promise in unsupervised stratification of disease trajectories. Yet, current approaches face significant challenges related to robustness, biomarker specificity, interpretability, and temporal resolution of clustering results. To address these challenges, we introduce Disease Progression Modeling and Stratification (DPMoSt), a new probabilistic model designed to optimize clusters of continuous trajectories along a long-term disease time axis. This approach allows for the determination of subtype-specific biomarkers, improving the accuracy of patient stratification and generalization on external cohorts. We demonstrate DPMoSt on both synthetic and real-world data for the modeling of Alzheimer’s disease (AD) evolution. In the synthetic experiments, DPMoSt shows high accuracy in reconstructing trajectory subtypes and identifying the biomarkers’ specificity for the clustering problem. Our experiments in the Alzheimer’s Disease Neuroimaging Initiative (ADNI) dataset demonstrate the ability of DPMoSt to identify AD subtypes associated with accelerated cognitive decline and higher prevalence of the APOE4 variants. This result was validated on the external memory clinic cohort of the Geneva University Hospitals, confirming the association between cognitive decline and APOE4 in the pathological subtype. These results highlight the robustness of DPMoSt as well as its potential for broader applicability, offering a powerful tool for studying disease progression and subtype differentiation across diverse populations.

## 1 Introduction

Disease Progression Modeling (DPM) aims at mathematically characterizing the evolution of the natural history of diseases from the analysis of patients’ cohorts. DPM holds the potential to provide relevant insights for improving our understanding of the disease, and identify novel treatment strategies (Khan & Iturria-Medina, 2024; Mould, 2012). However, defining computational models of the natural history of neurodegenerative disorders poses important challenges due to the significant heterogeneity in the disease trajectories observed between individuals. In fact, although the degenerative process may be similar, symptoms manifestation and biomarker profiles can vary significantly between patients due to factors such as demographics, genetic conditions, and lifestyle (Yang et al., 2011). To address this complexity, researchers are increasingly focusing on characterizing disease subtypes according to their phenotypic variability (Greenland et al., 2019).

In neuroscience and neurodegeneration research, innovative methodologies have been developed in the past years to automatically identify subtypes in disease progression. SuStaIn is a state-of-the-art unsupervised learning approach that identifies subpopulations and their corresponding trajectories within datasets of patients and control groups (Young et al., 2018, 2021, 2023). This method has been demonstrated in various clinical applications, allowing the detection of patterns of biomarker changes within subpopulations (Aksman et al., 2021; Zhou et al., 2023). Nevertheless, we acknowledge key limitations of this approach affecting robustness and interpretability of the subtyping results. First, in its standard form, SuStaIn models disease progression as a series of discrete events, thus limiting the temporal granularity and subsequent interpretability of the clustering results. Second, the progressions of temporal events are determined by cutoff values for biomarker separation between normal and pathological groups, which may not faithfully represent the real-world biomarkers’ distribution and thus affect the robustness of the clustering solution. This problem is particularly relevant when it comes to establishing the specificity of biomarkers between subtypes. In this case, we lack a sound quantification of the uncertainty of biomarkers trajectories and of their relative importance to determine the progression of specific disease subtypes, ultimately affecting the interpretability and robustness of SuStaIn’s findings. Finally, the standard SuStaIn implementation relies on cross-sectional data analysis, which overlooks temporal correlations in longitudinal datasets. As a consequence, the method may struggle to detect subtle biomarker variations over time, potentially compromising its accuracy in measuring individual disease progression and pathological changes. Although the recent work (Young et al., 2023) proposed to account for serial measurements in SuStaIn, disease trajectories are still based on discrete events and there is no quantification of biomarkers’ specificity, thus making this approach suboptimal for the quantification of subtle temporal changes in patients.

Leaspy proposes an alternative approach to the trajectory clustering problem (Poulet & Durrleman, 2021). Differently from SuStaIn, this approach relies on continuous parametric models to estimate disease trajectories while accounting for individual random effects through time-warp functions (Schiratti et al., 2017). However, similarly to SuStaIn, this approach relies on the assumption that all biomarkers must follow distinct sub-progressions, that is, that all biomarkers are equally important in determining the sub-trajectories; the lack of quantification of the biomarkers specificity critically affects the possibility to quantify the specificity of sub-progression and robustly characterize disease subtypes.

Other general clustering methods for patients’ trajectories have been proposed in the machine learning community, based on the estimation of latent representations of the patient variability. For example, the approach proposed in Chen et al. (2022) tackles the sub-typing problem by estimating the number of subtrajectories a posteriori through k-means clustering of patients’ representation in a latent space. We note that this method is not designed to model uncertainty or variability in possible trajectory configurations, potentially oversimplifying the underlying heterogeneity present in the disease process. In addition, the model potentially requires long follow-up data per patient to capture a meaningful latent representation through the proposed recurrent architecture. This aspect may, therefore, lead to suboptimal results when applied to short-term time series typical of neurodegeneration research. The approach (Lee & Van Der Schaar, 2020) extends the concept of latent subtyping by optimizing the clustering problem in the latent space at training time. Nevertheless, similarly to Leaspy, the latent representation does not allow to identify the specificity of the biomarkers in determining the subtypes. Moreover, the optimization problem is solved by predicting a given outcome of interest at each time stamp (e.g., diagnosis). The supervised setting of this method sets it apart from general DPM, in which we are more interested in describing and predicting the evolution of biomarkers across the disease timeline.

To address these limitations, we introduce Disease Progression Modeling and Stratification (DPMoSt), a new DPM framework designed to enhance sensitivity in capturing sub-types in disease progression. DPMoSt relies on a continuous model of disease trajectory, in which short-term observations across patients are modeled to estimate disease progression on a long-term time scale. Unlike existing approaches that optimize a global trajectory common to all biomarkers, DPMoSt quantifies the specificity of each biomarker in defining subtype trajectories. This allows for a more detailed and clinically interpretable understanding of disease progression.

We tested DPMoSt on a large set of synthetic data and on the problem of disease subtyping in ADNI and on the Geneva memory clinic cohort of the Geneva University Hospitals (GMC). The synthetic results demonstrate that DPMoSt effectively models biomarker trajectories and captures biomarker specificity in determining subtypes and their partitions. When applied to the data from the Alzheimer’s Disease Neuroimaging Initiative (ADNI), DPMoSt successfully reconstructs disease trajectories consistent with the amyloid cascade hypothesis (Jack et al., 2010; Karran et al., 2011). In particular, the trajectories subtypes are associated with varying rates of cognitive decline, and the most pathological progression presents a significantly higher prevalence of APOE4. This finding was validated on the data of the memory clinic cohort of the GMC, in which we could replicate the association between APOE4 prevalence and pathological subtypes. Notably, while competing trajectory clustering methods from the state-of-the-art could identify subtypes associated with accelerated cognitive decline in ADNI, the resulting biomarker trajectories did not generalize to the GMC testing cohort.

These results demonstrate DPMoSt’s ability to detect subtle biomarker changes linked to disease subtypes, making it a promising tool for advancing our understanding of AD, improving patient staging, and guiding clinical interventions.

The manuscript is structured as follows: Section 2 details the mathematical formulation of the model explaining key design choices and their rationale. Section 3 presents the experimental setting using both synthetic and ADNI dataset as well as an external cohort as a test dataset. Finally, Section 4 discusses the obtained results providing insights into the practical applicability of the model.

## 2 Methods

In this section, we formulate the modeling paradigm of DPMoSt and derive the related optimization procedure.

DPMoSt builds upon the established framework of self-modeling regression (Coull & Staudenmayer, 2004; Donohue et al., 2014; Lawton et al., 1972). For each individual j and biomarker b, we define the time-series measurements as ${\text{x}}_{j}^{b}=(x_{j}^{b}(t_{j}^{1}),\ldots ,x_{j}^{b}(t_{j}^{h_{j}}))$ , where, without loss of generality and for notational convenience, we assume that the sampling times are common across all biomarkers. We furthermore define the complete collection of measurements across all J subjects and B biomarkers as $\text{x}={\left({\left({\text{x}}_{b}^{j}\right)}_{j=1}^{J}\right)}_{b=1}^{B}$.

We aim at reparameterizing the individual longitudinal data with respect to an absolute disease time scale, allowing us to consider the biomarker’s evolution in relation to the progression of the pathology. To this end, we transform the set of individual’s time points $\left(t_{j}^{1},\ldots ,t_{j}^{h_{j}}\right)$ through a subject-specific time re-parametrization function $\phi_{j}(t_{j}^{\ell })$ . Typical transformations proposed in the literature are based either on translations or linear mappings (Donohue et al., 2014; Jedynak et al., 2015; Schiratti et al., 2015), and in what follows we consider a re-parametrization of the form $\phi_{j}(t_{j}^{\ell })=t_{j}^{\ell }+\tau_{j}$, where $\tau_{j}$ is a subject-specific translation factor that applies to each individual time point $t_{j}^{\ell }$, for $\ell =1,\ldots ,h_{j}$ across biomarkers (Li et al., 2018; Lorenzi et al., 2017). This term quantifies the individual disease severity along the disease time scale, allowing us to obtain a common representation of the individual observations along the long-term trajectory of disease progression.

We furthermore assume that the biomarkers’ trajectories can be described by a Sigmoid functional $S(\cdot |\bar{\theta })$ encoding a monotonically increasing process representing the transition from healthy to pathological stages. The parameters $\bar{\theta }$ represent the midpoint, rate of growth, and supremum of the Sigmoid. Finally, we incorporate additive Gaussian noise with a standard deviation $\sigma ={\left(\sigma_{b}\right)}_{b=1}^{B}$, where each $\sigma_{b}$ is biomarker specific.

Our goal is to model the trajectories associated with disease subtypes along with their specific set of biomarkers. To this end, we consider a two-level mixture model (McLachlan et al., 2019) to identify subtypes and their respective subdivisions. More precisely, the first level of the mixture decomposes the group-wise biomarker trajectory as the combination of multiple sub-trajectories associated with distinct subtypes. The second level of the mixture model applies when the biomarker trajectory is parametrized by the mixture of Sigmoids and considers that each subject j belongs to a given Sigmoid sub-trajectory.

Assuming independence among subjects and biomarkers, we can decompose the likelihood function to emphasize the two-tiered mixture model structure. In this formulation, the first level deals with the subtypes inference task, while the second one determines the probability of a subject to belong to a particular subtype:

$$p(\text{x}|\theta ,\sigma ,\xi ,\pi ,\tau )=\prod_{j,b}\left[\sum_{m=1}^{M}\xi_{b}^{m}\sum_{k=1}^{m}\pi_{j}^{\left(m,k\right)}p\left({\text{x}}_{j}^{b}|{\bar{\theta }}_{b}^{\left(m,k\right)},\sigma_{b},\tau_{j}\right)\right],$$ (1)

where the density of each likelihood for a given subject and biomarker is given by the additive Gaussian noise and independence assumptions

$$\begin{array}{c} \begin{array}{c} p({\text{x}}_{j}^{b}|{\bar{\theta }}_{b}^{\left(m,k\right)},\sigma_{b},\tau_{j})=\prod_{\ell =1}^{h_{j}}\text{NormPDF}\;\;(x_{j}^{b}(t_{j}^{\ell }),S(\phi (t_{j}^{\ell })|{\bar{\theta }}_{b}^{\left(m,k\right)}),\sigma_{b}^{2}). \end{array} \end{array}$$ (2)

**Fig. 1.**
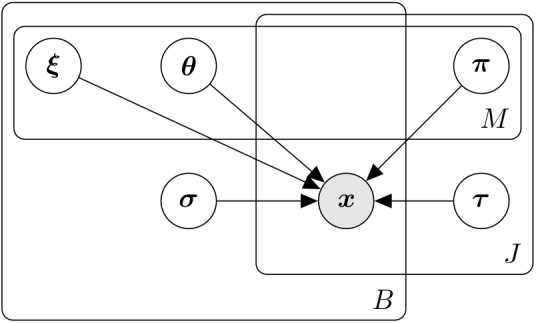
Graphical model for the DPMoSt two-level mixture model. We indicate with M the number of allowed subtypes, B the number of biomarkers, and J the total number of subjects.

With reference to Figure 1, in the general case in which we consider M sub-trajectories, the model is parameterized by the Sigmoids parameters for all biomarkers and all possible mixtures given by $\theta =\;\cup_{1\leq m\leq M}\theta^{m},$ where $\theta^{m}={\left({\bar{\theta }}_{b}^{\left(m,1\right)},\ldots ,{\bar{\theta }}_{b}^{\left(m,m\right)}\right)}_{b=1}^{B}$; the Sigmoid mixture coefficients defining the specificity of the biomarkers, for the first level of the mixture model $\xi ={\left(\xi_{b}^{0},\ldots ,\xi_{b}^{M}\right)}_{b=1}^{B}$; and the mixture coefficients for the second level of the mixture model $\pi =\cup_{1\leq m\leq M}\pi^{\ell },$ where $\pi^{m}={\left(\pi_{j}^{\left(m,1\right)},\ldots ,\pi_{j}^{\left(m,m\right)}\right)}_{j=1}^{J}$. Moreover, we have the following constraints on the mixture coefficients: $\sum_{m}\xi_{b}^{m}=\sum_{\ell }\pi_{j}^{\left(m,\ell \right)}=1\;\;\forall b,j,m$ . For example, if we consider M=2 , the progression associated with biomarker b can be explained by a single Sigmoid function with parameters ${\bar{\theta }}_{b}^{\left(1,1\right)}$ or by the mixture of two independent Sigmoids parametrized by ${\bar{\theta }}_{b}^{\left(2,1\right)}$ and ${\bar{\theta }}_{b}^{\left(2,2\right)},$ respectively. These two different configurations, single versus mixture of Sigmoids, occur with associated probability $\xi_{b}^{1}$ and $\xi_{b}^{2}=1-\xi_{b}^{1},$ respectively. The second level of the mixture model applies when the biomarker trajectory is parametrized by the mixture of Sigmoids and considers that each subject j belongs to the first Sigmoid of the mixture with parameters ${\bar{\theta }}_{b}^{\left(2,1\right)}$ with probability $\pi_{j}^{\left(2,1\right)}$ and to the one parameterized by ${\bar{\theta }}_{b}^{\left(2,2\right)}$ with probability $\pi_{j}^{\left(2,2\right)}=1-\pi_{j}^{\left(2,1\right)}$.

We chose to adopt this notation for the Sigmoid parameters rather than a simple list representation, because each $\theta^{m}$ is itself a structured object—a vector containing m components $\left({\bar{\theta }}_{1}^{m},\ldots ,{\bar{\theta }}_{m}^{m}\right)$ , where each $\theta_{i}^{m}$ represents the probability of a specific split occurring at the level of complexity of m possible subtypes; this hierarchical formulation allows us to capture both the number of sub-trajectories and their associated branching probabilities in a compact and interpretable way.

We note that in our setting, both the Sigmoid mixture probabilities and the subject mixture probabilities are considered to be time independent. This allows our model to account for longitudinal data information, while ensuring that the probability of a subject belonging to a given subtype remains consistent across all biomarkers.

Given the model outlined above, the posterior distribution is given by

$$\begin{array}{c} \begin{array}{c} p(\theta ,\sigma ,\xi ,\pi ,\tau |\text{x})\propto p(\theta ,\sigma ,\xi ,\pi ,\tau )p(x|\theta ,\sigma ,\xi ,\pi ,\tau ), \end{array} \end{array}$$ (3)

where θ represents the collection of all Sigmoid parameters, ξ and π represent the collection of all mixture coefficients, while the conditioning on the time points has been omitted for simplicity of notation.

### 2.1 Prior distribution

As a consequence of the independence assumption among different parameters, subjects and biomarkers, we can factorize the prior distribution into the product of independent priors for each parameter:

$$\begin{array}{l} p(\theta ,\sigma ,\xi ,\pi ,\tau )=p(\theta )p(\sigma )p\left(\xi \right)p(\pi )p(\tau )\\ \;\;\;\;\;\;\;\;\;\;\;\;\;\;\;\;\;\;\;\;\;\;\;\;\;\;\;\;\;\;\;\;\;\;\;\;\;=p(\theta )\prod_{b}\left(p(\sigma_{b})\prod_{m}p\left(\xi_{b}^{m}\right)\right)\;p(\pi )\prod_{j}p(\tau_{j}), \end{array}$$ (4)

where we rely on the following assumptions:

- p(θ)∝1 and p(π)∝1 as improper uniform prior (Taraldsen et al., 2022) for the Sigmoid parameters and sub-population subdivision in order to encode lack of information;
- $p(\sigma_{b})∼\text{InvGam}(\beta_{\sigma }-1,\beta_{\sigma })$ is an Inverse Gamma distribution of shape parameter $\beta_{\sigma }-1$ and scale parameter $\beta_{\sigma }>1$ as classical prior distribution for the noise standard deviation (Calvetti et al., 2009) in order to penalize small values;
- $p(\xi_{b}^{m})∼\text{Laplace}(1,1/\beta_{\xi }^{m})$ is a Laplace distribution (Kaban, 2007) of location 1 and scale parameter $1\;/\;\beta_{\xi }^{m}$ restricted to the interval [0,1] with $\beta_{\xi }^{m}$ decreasing in m, as regularization term for penalizing the introduction of a sub-trajectory to prevent overfitting;
- $p(\tau_{j})∼N(0,1\;/\;\beta_{\tau })$ is a standard Gaussian distribution, as regularization term to prevent overfitting due to excessive time shifts.

We underline that despite these prior parameters $\beta_{\xi }^{m}$ do not depend on the number of observations, when the amount of available data is limited, DPMoSt tends to favor a simpler explanation of biomarker progression, for instance resorting to a single trajectory with a relatively high noise level to accommodate all observations. Conversely, when a larger volume of data is available, the model is better equipped to detect and support the presence of multiple subtrajectories, allowing for a more nuanced representation of disease heterogeneity. Furthermore, we chose to place a Laplace prior over the parameter $\xi_{b}$, which defines the specificity of the biomarker, instead of using a Dirichlet prior, because the Laplace prior introduces a well-known and interpretable L1 regularization effect; this regularization promotes sparsity in the split probabilities, allowing the model to favor simpler explanations when appropriate. Additionally, the strength of this regularization is governed by a single scale parameter, which we found to be easier to tune and interpret in terms of its influence on the resulting distribution.

### 2.2 Two-level expectation maximization

The number of parameters of the resulting model linearly scales with the number of subjects and biomarkers in the dataset. Specifically, for each Sigmoid function, we have three parameters ${\bar{\theta }}^{\left(m,k\right)}$ (midpoint, rate of growth, and supremum), and when considering M subtypes, this results in 3M(M+1) / 2 parameters for each biomarker. Furthermore, we consider for each biomarker a parameter for the noise standard deviation ($\sigma_{b}$), and M probabilities of subtype splitting ($\xi_{b}^{m}$). In addition, for each subject, we account for the assignment probabilities ($\pi_{j}^{\left(m,k\right)}$) for a total of M(M+1) / 2 , and parameter for the time-shift evaluation ($\tau_{j}$). Summing up, the total number of parameters of the model is B(3M(M+1)/2+2)+J(M(M+1)/2+1), where M is the number of sub-types, B is the number of biomarkers, and J is the number of subjects.

To limit the computational cost, we do not attempt to approximate the entire posterior distribution (3). Instead, we focus on obtaining point estimates by employing Maximum a Posteriori (MAP) estimation. Specifically, we use an Expectation-Maximization (EM) algorithm, which takes advantage of the two-level mixture structure of the model, performing iteratively the optimization of the parameters θ, σ, and τ via Stochastic Gradient Descent (SGD) and a classical EM step for the parameters ξ and π.

Thanks to the proposed formulation, DPMoSt is designed to offer interpretable insights into disease progression dynamics: first, it estimates the parameters governing the continuous trajectories of biomarkers; next, it calculates the probability that each biomarker can be split in distinct sub-trajectories; and finally, it determines the probability for each subject to belong to a specific sub-population.

#### 2.2.1 Example: Model with two sub-trajectories

In this section, we derive the equations for the particular case where the model considers two sub-trajectories, that is, M=2 . In this scenario, the general likelihood formulation given in Equation 1 simplifies, allowing for a more explicit representation of the underlying probability structure. Specifically, we show how the likelihood expression adapts when only two sub-trajectories contribute to the model:

$$\begin{array}{c} p\left(x|\theta ,\sigma ,\xi ,\pi ,\tau \right)=\prod_{j,b}[\xi_{b}^{1}p\left(x_{j}^{b}|{\bar{\theta }}_{b}^{\left(1,1\right)},\sigma_{b},\tau_{j}\right)+\\ \;(1-\xi_{b}^{1})\left(\pi_{j}^{\left(2,1\right)}p\left(x_{j}^{b}|{\bar{\theta }}_{b}^{\left(2,1\right)},\sigma_{b},\tau_{j}\right)+\left(1-\pi_{j}^{\left(2,1\right)}\right)p({\text{x}}_{j}^{b}|{\bar{\theta }}_{b}^{\left(2,2\right)},\sigma_{b},\tau_{j})\right)]. \end{array}$$ (5)

Within this formulation, the MAP estimate of the model, as defined by Equation 6, can be approximated following the procedure outlined in Algorithm 1.

$$\begin{array}{l} \hat{\theta },\hat{\sigma },\hat{\xi },\hat{\pi },\hat{\tau }=\text{arg}\;\text{max}\;\;\text{ln}(p(\theta ,\sigma ,\xi ,\pi ,\tau |\text{x}))\\ \;\;\;\;\;\;\;\;\;\;\;\;\;\;\;\;\;\;\;\;\;\;\;\;\;\;\;=\text{arg}\;\text{max}\;\;\text{ln}(p(x|\theta ,\sigma ,\xi ,\pi ,\tau ))-\beta_{\sigma }\sum_{b}\left(\text{In}(\sigma_{b})+\frac{1}{\sigma_{b}}\right)+\beta_{\xi }\sum_{b}\xi_{b}^{1}-\beta_{\tau }\sum_{j}\tau_{j}^{2}, \end{array}$$ (6)

where $\beta_{\xi }=1\;/\;\beta_{\xi }^{2}-1\;/\;\beta_{\xi }^{1}$. Moreover, we note that since we introduced a non-constant prior over the parameter ξ, it is necessary to choose $\beta_{\xi }$ in a way that ensures ξ as valid mixture coefficients in our model, that is, constrained between 0 and 1. In the Supplementary Material, we demonstrate that a sufficient condition is provided by $0<\beta_{\xi }<J$ , where J represents the number of subjects.

**Algorithm 1.** Disease Progression Modeling and Stratification (DPMoSt) for two sub-trajectories.

Initialize: $\hat{\xi }=\xi$ , $\hat{\pi }=\pi$ , $\hat{\theta }=\theta$ , $\hat{\sigma }=\sigma$ , $\hat{\tau }=\tau$ ;**for** *k* *=* *1* **to** *K-1* **do** ** for** *b* *=* *1* **to** *B* **do**; /* EM for subtype detection */ ** **${\hat{\xi }}_{b}^{1}=\frac{1}{J+({\hat{\xi }}_{b}^{1}-1)\beta_{\xi }^{1}}\sum_{j}p({\text{x}}_{j}^{b}|{\bar{\theta }}_{b}^{\left(1,1\right)}){\hat{\xi }}_{b}^{1}\;/\;p({\text{x}}_{j}^{b});$ ** end** ** for** *j* *=* *1* **to** *J* **do**; /* EM for subtype partition */ ** **${\hat{\pi }}_{j}^{\left(2,1\right)}=\frac{1}{J}\sum_{b}p({\text{x}}_{j}^{b}|{\bar{\theta }}_{b}^{\left(2,1\right)}){\hat{\pi }}_{j}^{\left(2,1\right)}\;/\;p({\text{x}}_{j}^{b});$ ** end** ** for** *i* *=* *1* **to** *N-1* **do** ** for** *j* *=* *1* **to** *J* **do**; /* Optimization for time-shift */ ** **${\hat{\tau }}_{j}={\hat{\tau }}_{j}-\lambda_{\tau }{▽}_{\tau_{j}}p(\hat{\theta },\hat{\sigma },\hat{\xi },{\hat{\pi }}_{j},{\hat{\tau }}_{j}|{\text{x}}_{j});$ ** end** ** for** *b* *=* *1* **to** *B* **do**; /* Optimization for Sigmoid parameters & noise std */ ** **${\hat{\theta }}_{b}={\hat{\theta }}_{b}-\lambda_{\theta }\nabla_{\theta_{b}}p({\hat{\theta }}_{b},{\hat{\sigma }}_{b},{\hat{\xi }}_{b},\hat{\pi },\hat{\tau }|{\text{x}}^{b});$ ** **${\hat{\sigma }}_{b}={\hat{\sigma }}_{b}-\lambda_{\sigma }\nabla_{\sigma_{b}}p({\hat{\theta }}_{b},{\hat{\sigma }}_{b},{\hat{\xi }}_{b},\hat{\pi },\hat{\tau }|{\text{x}}^{b});$ ** end** ** end** **end** **return** $\hat{\theta },\hat{\sigma },\hat{\xi },\hat{\pi },\hat{\tau };$

## 3 Results

In this section, we present the results of applying DPMoSt to both synthetic and real-world data, the latter represented by longitudinal multimodal markers for the Alzheimer’s Disease Neuroimaging Initiative (ADNI) and for the memory clinic cohort of the GMC. We also compare these results with those obtained using two well-established methods: Leaspy and SuStaIn. The validation on synthetic data allows for the comparison against a known ground truth, ensuring that the approach is effective in controlled conditions. The demonstration on real-world data offers instead a practical assessment of the model’s performance in relevant clinical scenarios.

### 3.1 Synthetic data

We assess the performance of the proposed method through a systematic evaluation across varying levels of data complexity. This involves altering key factors such as the number of biomarkers, the number of time points per subject, and the signal-to-noise ratio (SNR). This rigorous testing framework allows us to evaluate the robustness and accuracy of the model under diverse, realistic, and progressively challenging conditions.

#### 3.1.1 Data generation

For each configuration, we generate 100 different datasets with the following specifications:

- cohort Size: 50 subjects;
- time points per subject: each subject has a varying number of time points (2, 5, and 7), randomly sampled from the time interval [0,20]. This variability simulates realistic longitudinal datasets with differing observation densities;
- SNR: three levels of noise variance are tested (0.7, 0.5, and 0.2), representing high, medium, and low noise levels to evaluate the model’s robustness in noisy settings;
- number of biomarkers: each subject has 2, 5, or 7 biomarkers, with 1, 3, and 4 biomarkers, respectively, designated as subtype specific. This setup ensures clear differentiation between subtype-specific progression trajectories;
- subtype distribution: subjects are evenly divided across subtypes to maintain balanced group sizes for unbiased inference;
- biomarker trajectories: progression is modeled using Sigmoid functions. The function parameters—midpoint, supremum, and growth rate—are sampled from Gaussian distributions. Constraints are applied to ensure a positive supremum and biologically plausible growth rates.

This experimental design enables a systematic evaluation over a total of 1800 simulations (100×2×3×3 ) of the proposed method under different conditions, including varying noise levels, differing biomarker sets, and a range of observation time points for each subject. The randomized sampling of parameters ensures robust testing across a wide spectrum of scenarios.

#### 3.1.2 Performance metrics

After optimization, we quantified the following performance metrics to validate the obtained results.

##### 3.1.2.1 Trajectory approximation metric

First of all, we validate the trajectory approximation considering the relative error between the estimated and true rate of growths weighted by the probability of subtype to be considered:

$$\begin{array}{cc} \begin{array}{cc} err\left(r_{b}^{\text{est}},r_{b}^{\text{true}}\right)= & \frac{\xi_{b}^{1}}{2}\left(\left|\frac{r_{b}^{0}-{\bar{r}}_{b}^{0}}{{\bar{r}}_{b}^{0}}\;\right|+\left|\frac{r_{b}^{0}-{\bar{r}}_{b}^{1}}{{\bar{r}}_{b}^{1}}\;\right|\right)+\frac{1-\xi_{b}^{1}}{2}\left(\left|\frac{r_{b}^{1}-{\bar{r}}_{b}^{0}}{{\bar{r}}_{b}^{0}}\;\right|+\left|\frac{r_{b}^{2}-{\bar{r}}_{b}^{1}}{{\bar{r}}_{b}^{1}}\;\right|\right). \end{array} & \end{array}$$ (7)

Here, $r_{b}^{\text{est}}=(r_{b}^{0},r_{b}^{1},r_{b}^{2})$ represents the growth rates for the single trajectory and the two sub-trajectories, respectively; similarly, $r_{b}^{\text{true}}=({\bar{r}}_{b}^{0},{\bar{r}}_{b}^{1})$ denotes the true growth rate values, with the convention that the two values are identical when no split occurs; additionally, $1-\xi_{b}^{1}$ represents the probability of a split occurring. This measure enables a proper evaluation of the method’s performance in approximating the growth rate of progression, the only parameter of the biomarker trajectory that is meaningful to assess. In contrast, other parameters, such as the midpoint and supremum, may be influenced by the rescaling of the time axis.

We note that this metric can be only computed for DPMoSt and Leaspy, since SuStaIn is an event-based model and lacks by construction the concept of growth rate or continuous trajectories that can be extrapolated from its output.

##### 3.1.2.2 Subtype detection metric

Secondly, we assess the algorithm’s ability to accurately detect biomarker subtypes. This is quantified by considering the percentage of biomarkers correctly estimated as subtype specific or not. In this context, the split probability for DPMoSt is defined as the maximum of the probabilities for a split occurring or not occurring. Although neither Leaspy nor SuStaIn directly provides biomarker-specific split probabilities, for the sake of comparison, we introduce a notion of biomarker specificity for these methods. More precisely, a biomarker is considered specific only if the sub-trajectories show significant differences in their progression. For SuStaIn, we declare two sequences of events associated with a biomarker to be distinct if the respective orderings differ for more than 10% of the total number of events. In Leaspy, we consider two biomarker trajectories to be different if their mean squared differences across the entire time axis are larger than twice the estimated standard deviation of the trajectories. These cut-off values were established based on preliminary simulations in order to maximize the accuracy in detecting the splits.

##### 3.1.2.3 Subtype partition metric

Lastly, we assess the accuracy of subtype partition detection. To this end, we compute the area under the curve (AUC) to quantify the model’s ability to assign subjects to the correct subtype. While DPMoSt and Leaspy inherently provide subject-specific subtype partitions, it is important to note that SuStaIn does not naturally offer subject-specific subtype assignments due to its reliance on cross-sectional data. To address this limitation, we consider two different approaches to assign a subject to a given subtype. The first approach consists in considering the subtype estimated for the subject at the last visit. The second approach is based on computing the majority subtype across all subject’s time points.

#### 3.1.3 Algorithm settings

We concentrate on the scenario where two subtypes, that is, M=2 , are taken into account. To avoid local maxima in the posterior distribution, we initialized the DPMoSt model as follows. We set $\xi_{b}^{1}=\xi_{b}^{2}=0.5$ and $\pi_{j}=0.5$ , reflecting a complete lack of prior information on the sub-trajectories and sub-population probabilities. The noise standard deviation was initialized to match the standard deviation of the data, and the parameters of the Sigmoid functions were randomly sampled from Gaussian distributions, ensuring a positive growth rate and supremum. Regarding the prior parameters, we set $\beta_{\xi }$ and $\beta_{\sigma }$ to 15% of the number of subjects; this choice strikes a balance between effective regularization and keeping values within a reasonable range.

To prevent the trajectories from becoming unrealistically stretched over time, we set the prior parameter $\beta_{\tau }=1/2\sigma_{\tau }^{2}$, where $\sigma_{\tau }=40$ . This effectively restricts the allowable time shift, expressed in months, to approximately ±10 years, which is a plausible and clinically meaningful range for the timeline of neurodegenerative processes.

For Leaspy (https://gitlab.com/icm-institute/aramislab/leaspy) and SuStaIn (https://github.com/ucl-pond/pySuStaIn), we followed the guidelines provided in their respective GitHub repositories. Both methods were constrained to consider two subtypes; after obtaining the solutions, we identified biomarkers that exhibited splitting behavior versus those that did not, using the procedure described above.

#### 3.1.4 Comparison DPMoSt Leaspy and SuStaIn

##### 3.1.4.1 Trajectory approximation

**Fig. 2.**
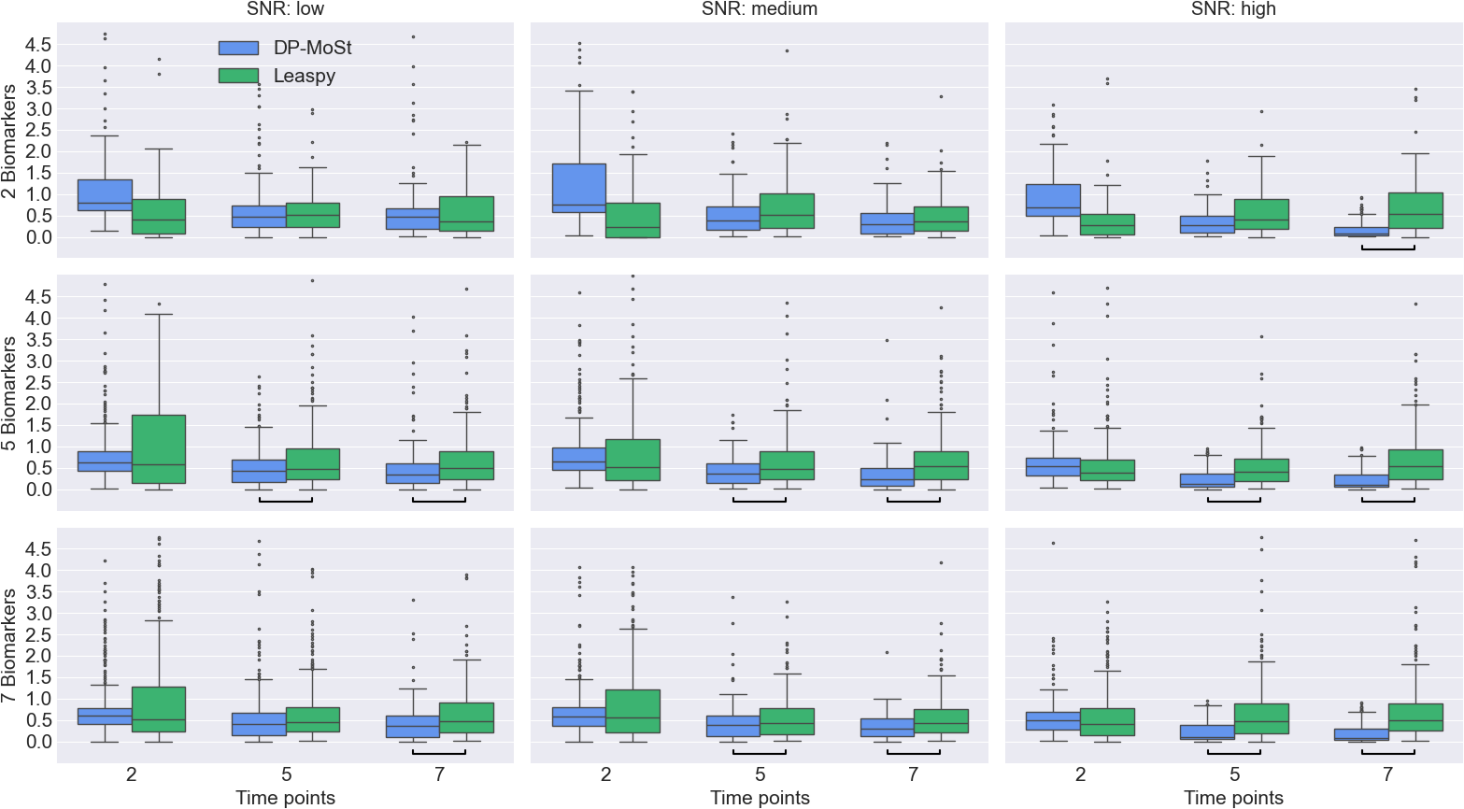
The figure illustrates the relative errors in approximating growth rates across various simulations, comparing DPMoSt (blue) and Leaspy (green). Results are organized by rows to reflect variations in the number of biomarkers and by columns to show variations in the number of time points per subject and the signal-to-noise ratio (SNR). The black bars at the bottom of the plot indicate statistical significance (p<0.01 , ANOVA test Bonferroni corrected for multiple comparisons). The figure highlights that DPMoSt outperforms Leaspy in terms of trajectory reconstruction across most configurations.

In Figure 2, we present the trajectory approximation error for both DPMoSt and Leaspy. As previously mentioned, a comparison with SuStaIn is not possible because this method is an event-based model, which approximates only a discrete series of events rather than a continuous trajectory.

The rows represent scenarios with an increasing number of biomarkers, while the columns correspond to scenarios with varying SNRs and number of time points for each subject. We observe that, in most cases, DPMoSt significantly outperforms Leaspy in trajectory reconstruction (p<0.05 , ANOVA test Bonferroni corrected for multiple comparisons), particularly in more complex models that include a greater number of biomarkers and additional time points.

##### 3.1.4.2 Subtype detection

Table 1 presents the mean accuracy of subtype detection across simulations for all three methods. To ensure a fair comparison, we constrained both Leaspy and SuStaIn to estimate two subtypes, as they cannot inherently detect biomarker specificity.

**Table 1.** The table presents the mean accuracy for subtype detection across various simulations, along with the corresponding standard deviations for the three algorithms, respectively DPMoSt, Leaspy, and SuStaIn.

		SNR: low			SNR: medium			SNR: high		
Biom.	T.P.	DPMoSt	Leaspy	SuStaIn	DPMoSt	Leaspy	SuStaIn	DPMoSt	Leaspy	SuStaIn
2	2	0.6 ($10^{-4}$ )	0.5 ($10^{-3}$ )	0.5 ($10^{-3}$ )	0.71 ($10^{-3}$ )	0.49 ($10^{-4}$ )	0.5 ($10^{-3}$ )	0.79 ($10^{-4}$ )	0.48 ($10^{-3}$ )	0.5 ($10^{-4}$ )
	5	0.83 ($10^{-3}$ )	0.47 ($10^{-3}$ )	0.5 ($10^{-3}$ )	0.85 ($10^{-4}$ )	0.52 ($10^{-3}$ )	0.5 ($10^{-4}$ )	0.93 ($10^{-4}$ )	0.47 ($10^{-3}$ )	0.5 ($10^{-4}$ )
	7	0.82 ($10^{-3}$ )	0.45 ($10^{-3}$ )	0.5 ($10^{-3}$ )	0.89 ($10^{-4}$ )	0.48 ($10^{-3}$ )	0.5 ($10^{-4}$ )	0.92 ($10^{-4}$ )	0.5 ($10^{-3}$ )	0.5 ($10^{-3}$ )
5	2	0.7 ($10^{-4}$ )	0.5 ($10^{-4}$ )	0.41 ($10^{-3}$ )	0.76 ($10^{-3}$ )	0.53 ($10^{-3}$ )	0.42 ($10^{-3}$ )	0.78 ($10^{-3}$ )	0.53 ($10^{-4}$ )	0.5 ($10^{-3}$ )
	5	0.77 ($10^{-4}$ )	0.51 ($10^{-3}$ )	0.76 ($10^{-3}$ )	0.84 ($10^{-3}$ )	0.51 ($10^{-3}$ )	0.75 ($10^{-3}$ )	0.9 ($10^{-4}$ )	0.53 ($10^{-3}$ )	0.75 ($10^{-4}$ )
	7	0.76 ($10^{-4}$ )	0.48 ($10^{-3}$ )	0.75 ($10^{-3}$ )	0.84 ($10^{-3}$ )	0.46 ($10^{-3}$ )	0.73 ($10^{-3}$ )	0.96 ($10^{-4}$ )	0.5 ($10^{-3}$ )	0.64 ($10^{-4}$ )
7	2	0.72 ($10^{-3}$ )	0.54 ($10^{-4}$ )	0.55 ($10^{-3}$ )	0.78 ($10^{-3}$ )	0.54 ($10^{-3}$ )	0.62 ($10^{-3}$ )	0.87 ($10^{-3}$ )	0.55 ($10^{-4}$ )	0.77 ($10^{-4}$ )
	5	0.71 ($10^{-3}$ )	0.51 ($10^{-3}$ )	0.85 ($10^{-3}$ )	0.87 ($10^{-3}$ )	0.49 ($10^{-3}$ )	0.87 ($10^{-3}$ )	0.94 ($10^{-4}$ )	0.5 ($10^{-3}$ )	0.88 ($10^{-4}$ )
	7	0.79 ($10^{-3}$ )	0.49 ($10^{-3}$ )	0.87 ($10^{-3}$ )	0.84 ($10^{-4}$ )	0.53 ($10^{-3}$ )	0.74 ($10^{-3}$ )	0.96 ($10^{-4}$ )	0.51 ($10^{-3}$ )	0.78 ($10^{-4}$ )

Values with a *p*-value of p<0.01 Bonferroni corrected for multiple comparisons are highlighted in bold. In the table, SNR denotes the signal-to-noise ratio, Biom. represents the number of biomarkers in the simulated configuration, and T.P. indicates the number of time points per subject in the dataset. The table shows that DPMoSt generally outperforms Leaspy and SuStaIn in terms of subtype detection accuracy.

For each biomarker, we determined the existence of the split based on the differences in trajectories across the identified subtypes; statistically significant differences between subgroups, determined using an ANOVA test (p<0.01 Bonferroni corrected for multiple comparisons), are highlighted in the figure.

The results demonstrate that DPMoSt significantly outperforms the other methods, with its performance improving as SNR increases and as more time points per subject are included. This trend is consistent for SuStaIn but less so for Leaspy, whose ability to distinguish subtypes is notably weaker than the other methods. The low performance of Leaspy in the subtype detection task may also explain its lower trajectory approximation results, where the tendency to overfit seems to limit the capacity to effectively model population subtypes.

These results indicate that DPMoSt is able to identify biomarker-specific subtypes, a capability not inherent to Leaspy or SuStaIn, even with adjustments.

##### 3.1.4.3 Subtype partition

Table 2 illustrates the mean accuracy of the subtype partitioning across simulations for all three methods. The results indicate that both DPMoSt and SuStaIn outperform Leaspy in subtype partitioning accuracy. Despite its weaker performance in subtype detection, SuStaIn demonstrates a more robust subtype partitioning capabilities, performing comparably with DPMoSt when the average subtype is considered, while performing slightly worse when the last subtype is considered.

**Table 2.** The table presents the mean accuracy for subtype partition across various simulations, along with the corresponding standard deviations for the three algorithms, respectively, DPMoSt, Leaspy, and SuStaIn.

		SNR: low				SNR: medium				SNR: high			
Biom.	T.P.	DPMoSt	Leaspy	SuStaIn (Last)	SuStaIn (AVG)	DPMoSt	Leaspy	SuStaIn (Last)	SuStaIn (AVG)	DPMoSt	Leaspy	SuStaIn (Last)	SuStaIn (AVG)
2	2	0.71 ($10^{-2}$ )	0.54 ($10^{-2}$ )	0.73 ($10^{-2}$ )	0.76 ($10^{-2}$ )	0.75 ($10^{-2}$ )	0.55 ($10^{-4}$ )	0.78 ($10^{-2}$ )	0.8 ($10^{-2}$ )	0.78 $\left(20^{-2}\right)$	0.56 ($10^{-2}$ )	0.86 $\left(20^{-2}\right)$	0.89 ($10^{-2}$ )
	5	0.9 ($10^{-2}$ )	0.58 ($10^{-2}$ )	0.72 ($10^{-2}$ )	0.85 ($10^{-2}$ )	0.91 $\left(20^{-2}\right)$	0.6 ($10^{-2}$ )	0.77 $\left(20^{-2}\right)$	0.92 ($10^{-2}$ )	0.97 ($10^{-4}$ )	0.63 ($10^{-2}$ )	0.83 $\left(20^{-2}\right)$	0.91 ($10^{-2}$ )
	7	0.94 ($10^{-2}$ )	0.61 ($10^{-2}$ )	0.74 ($10^{-2}$ )	0.91 ($10^{-2}$ )	0.95 $\left(20^{-2}\right)$	0.62 ($10^{-2}$ )	0.75 $\left(20^{-2}\right)$	0.91 ($10^{-2}$ )	0.97 ($10^{-4}$ )	0.66 ($10^{-2}$ )	0.83 ($10^{-2}$ )	0.93 ($10^{-2}$ )
5	2	0.88 $\left(20^{-2}\right)$	0.56 ($10^{-4}$ )	0.89 ($10^{-2}$ )	0.93 ($10^{-2}$ )	0.94 ($10^{-2}$ )	0.58 ($10^{-2}$ )	0.88 ($10^{-2}$ )	0.91 ($10^{-2}$ )	0.92 ($10^{-2}$ )	0.58 ($10^{-4}$ )	0.93 ($10^{-2}$ )	0.94 ($10^{-2}$ )
	5	0.97 ($10^{-4}$ )	0.61 ($10^{-2}$ )	0.92 ($10^{-2}$ )	0.98 ($10^{-2}$ )	0.98 ($10^{-2}$ )	0.6 ($10^{-2}$ )	0.94 ($10^{-2}$ )	0.98 ($10^{-2}$ )	0.99 ($10^{-4}$ )	0.59 ($10^{-2}$ )	0.98 ($10^{-4}$ )	1 ($10^{-4}$ )
	7	0.98 ($10^{-4}$ )	0.58 ($10^{-2}$ )	0.9 ($10^{-2}$ )	0.99 ($10^{-2}$ )	0.98 ($10^{-2}$ )	0.61 ($10^{-2}$ )	0.95 ($10^{-2}$ )	1 ($10^{-4}$ )	1 ($10^{-4}$ )	0.64 ($10^{-2}$ )	0.98 ($10^{-4}$ )	1 ($10^{-4}$ )
7	2	0.92 ($10^{-2}$ )	0.58 ($10^{-4}$ )	0.95 ($10^{-2}$ )	0.97 ($10^{-2}$ )	0.95 ($10^{-2}$ )	0.59 ($10^{-2}$ )	0.98 ($10^{-2}$ )	0.99 ($10^{-2}$ )	0.97 ($10^{-2}$ )	0.59 ($10^{-4}$ )	0.99 ($10^{-4}$ )	0.99 ($10^{-4}$ )
	5	0.96 ($10^{-2}$ )	0.61 ($10^{-2}$ )	0.96 ($10^{-2}$ )	1 ($10^{-4}$ )	0.99 ($10^{-2}$ )	0.58 ($10^{-2}$ )	0.99 ($10^{-2}$ )	1 ($10^{-4}$ )	1 ($10^{-4}$ )	0.59 ($10^{-2}$ )	1 ($10^{-4}$ )	1 ($10^{-4}$ )
	7	0.98 ($10^{-2}$ )	0.63 ($10^{-2}$ )	0.95 ($10^{-2}$ )	1 ($10^{-4}$ )	1 ($10^{-4}$ )	0.61 ($10^{-2}$ )	0.96 ($10^{-2}$ )	1 ($10^{-4}$ )	1 ($10^{-4}$ )	0.64 ($10^{-2}$ )	1 ($10^{-4}$ )	1 ($10^{-4}$ )

Values with an adjusted *p*-value of p<0.01 are highlighted in bold. In the table, SNR denotes the signal-to-noise ratio, Biom. represents the number of biomarkers in the simulated configuration, and T.P. indicates the number of time points per subject in the dataset. The table shows that DPMoSt achieves subtype partitioning performance that outperforms the one of Leaspy and is comparable with, and occasionally surpasses, the one of SuStaIn.

Overall, these findings emphasize that DPMoSt not only performs well in continuous trajectory estimation and subtype detection but also achieves subtype partitioning performance that is comparable with, and occasionally surpasses, the one of SuStaIn.

### 3.2 ADNI dataset

We present here the results obtained applying DPMoSt to the ADNI dataset.

The Alzheimer’s Disease Neuroimaging Initiative (ADNI) database (adni.loni.usc.edu) was established in 2003 as a public–private partnership under the leadership of Principal Investigator Michael W. Weiner, MD (a complete listing of ADNI investigators can be found at: http://adni.loni.usc.edu/wp-content/uploads/how_to_apply/ADNI_Acknowledgement_List.pdf). Initially, ADNI aimed to determine whether serial magnetic resonance imaging (MRI), positron emission tomography (PET), other biological markers, and clinical and neuropsychological assessments could be combined to track the progression of Mild Cognitive Impairment (MCI) and early Alzheimer’s disease (AD). Today, its objectives have expanded to include validating biomarkers for clinical trials, enhancing the diversity of the participant cohort to improve the generalizability of its data, and providing valuable resources on the diagnosis and progression of Alzheimer’s disease to the scientific community.

In this study we considered the ADNIMERGE data (The ADNI Team, 2023), which is a comprehensive dataset created by merging several key tables from ADNI1 to ADNI3 databases. To ensure clinical homogeneity and relevance of the study population with respect to the pathological trajectory of Alzheimer’s disease, we focused on subjects with pathological baseline Cerebrospinal Fluid (CSF) Aβ amyloid levels below the threshold of 192 pg/ml. Our cohort thus consisted of 452 subjects classified as 113 Clinically Normal (CN), 30 Subjective Memory Complaint (SMC), 103 Early Mild Cognitive Impairment (EMCI), 126 Late Mild Cognitive Impairment (LMCI), and 80 Alzheimer’s disease (AD). Table 3 reports summary sociodemographic and clinical information for the selected individuals.

**Table 3.** Baseline sociodemographic and clinical information for ADNI study cohort.

Group	CN	SMC	EMCI	LMCI	AD
N subjects	113	30	103	126	80
Age (years)	75 (5)	72 (5)	71 (7)	74 (8)	74 (8)
Gender (% F)	55	63	52	33	43
Education (years)	16 (3)	17 (2)	15 (2)	16 (3)	15 (3)
Hippocampus (${\text{cm}}^{3}$)	7.3 (0.9)	7.7 (0.8)	7.2 (1.0)	6.5 (1.1)	5.9 (1.1)
Ventricles (${\text{cm}}^{3}$)	33.5 (18.3)	31.2 (18.7	34.6 (20.3)	47.2 (26.9)	50.2 (27.7)
Entorhinal (${\text{cm}}^{3}$)	3.8 (0.6)	4.1 (0.7)	3.7 (0.7)	3.4 (0.8)	2.9 (0.7)
WholeBrain (${\text{cm}}^{3}$)	1019 (105)	1060 (96)	1058 (106)	1031 (102)	1060 (96)
ADAS11	6.8 (5.7)	4.8 (2.7	8.7 (6.2)	14.4 (8.2)	21.7 (8.6)
FAQ	1.7 (5.4)	0.6 (1.3)	2.9 (5.3)	7.7 (8.4)	15.9 (7.4)
MOCA	25 (3)	26 (2)	24 (3)	21 (4)	18 (3)
AV45	1.1 (0.2)	1.2 (0.2	1.2 (0.2)	1.3 (0.2)	1.4 (0.2)
FDG	1.3 (0.1)	1.3 (0.1)	1.3 (0.1)	1.1 (0.1)	1.0 (0.1)
MMSE	29 (2)	29 (1)	28 (3)	25 (4)	21 (4)
CDRSB	0.7 (2.4)	0.2 (0.4)	1.5 (1.7)	3.1 (3.2)	5.5 (2.9)

CN: Cognitively Normal; SMC: Subjective Memory Complaints; EMCI: Early Mild Cognitive Impairment; AD: Alzheimer’s disease; LMCI: Late Mild Cognitive Impairment.

#### 3.2.1 Data analysis

Our analysis is based on standard clinical and imaging measures to map the progression of AD across clinical stages (Budelier & Bateman, 2020; Jack et al., 2010; Karran et al., 2011). More precisely we considered volumetric measures (hippocampal, ventricular, and entorhinal volumes); glucose metabolism (average normalized FDG uptake in the same regions); brain amyloidosis (average normalized AV45 uptake in the frontal cortex, anterior cingulate, precuneus, and parietal cortex); and functional and neuropsychological scores (ADAS11, FAQ, MOCA, MMSE, and CDRSB). Volumetric measures were scaled by each individual’s total intracranial volume.

The long-term disease progression was obtained by estimating a reparametrized timeline axis and by fitting a monotonically increasing Sigmoid function to capture the gradual degeneration of each individual trajectory from healthy to pathological stages. Trajectory clustering was performed by analyzing potential variations in the modeled trajectories, providing a probability estimate of whether a biomarker exhibits evidence of different progressions across subtypes. We initialize the three algorithms in the same way as described in Section 3.1.3.

#### 3.2.2 DPMoSt results

##### 3.2.2.1 Subtypes inference

**Fig. 3.**
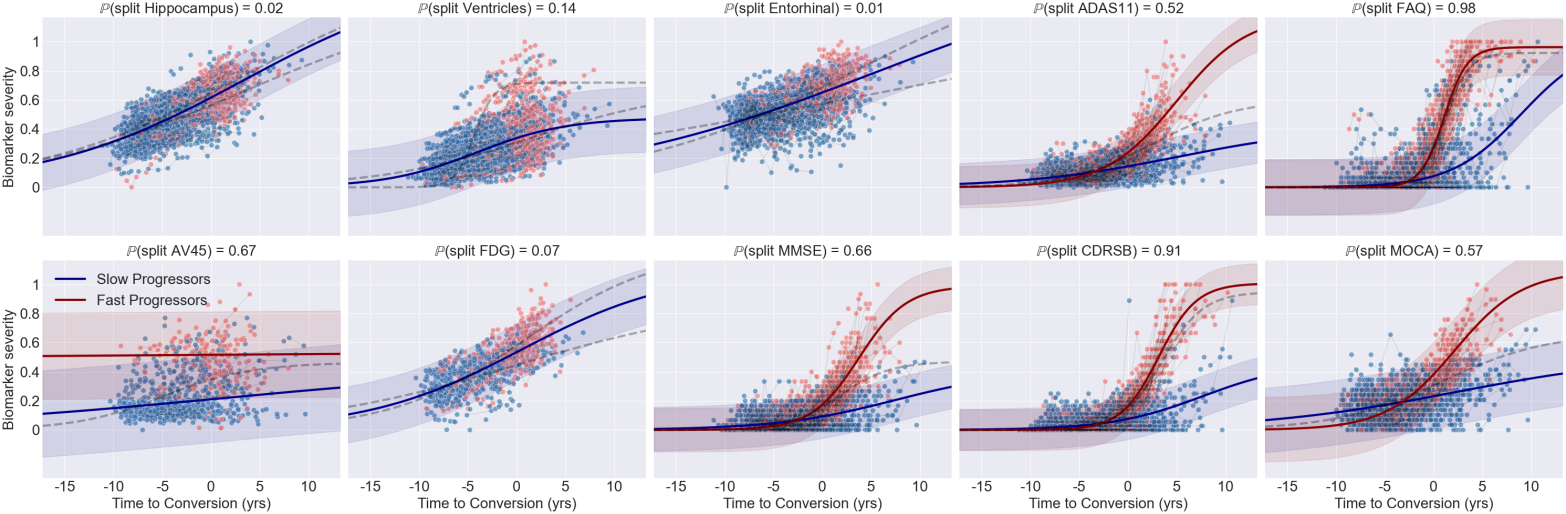
The figure shows the biomarker trajectories estimated by DPMoSt on the ADNI dataset. The y-axis represents the biomarker severity, and the x-axis denotes the reparametrized time. Dots represent individual longitudinal measurements for each subject, while solid lines represent the estimated trajectories with shaded bands indicating standard deviation. Colors differentiate the two subtypes identified by the model. In each plot title, we provide the probability associated with the biomarker specificity for the subtype. The results highlight the importance of ADAS11, FAQ, AV45, MMSE, CDRSB, and MOCA in defining the subtypes.

Figure 3 illustrates the results obtained by applying DPMoSt to the study population. The x-axis represents the time to conversion (in years), while the y-axis represents biomarker severity as z-scores, obtained after normalization.

Our analysis identifies two distinct trajectory subtypes (blue and red curves) associated with 225 and 227 subjects. The trajectories are significantly different with respect to the evolution of clinical scores, whereby the fast progressors (red) are associated with earlier progression onset and speed than the slow progressors (blue).

DPMoSt identifies three broad typologies of markers: (1) *specific*, such as neuropsychological scores (FAQ, MMSE, and CDRSB) and amyloid load (AV45), where the model reveals two clearly distinct trajectories with high probability; (2) *moderately specific*, including ventricular volume, ADAS11 and MOCA, where the splitting of the trajectory in two subtypes has not negligible probability; and (3) *non-specific*, such as hippocampal, entorhinal volumes, and FDG uptake, for which the model suggests a unique trajectory across subtypes.

Focusing on the most distinctive markers, we note that FAQ and CDRSB have a steeper increase roughly 3 years before the slow progressors. Another distinguishing feature is the global amyloid level measured by AV45, which is consistently higher for the fast progressors. We can furthermore observe that AV45 levels show relatively constant evolution, likely reflecting the pathological Aβ CSF amyloid levels of the study population at baseline. We note that the actual split is not necessarily related to the absolute strength of the difference between the distributions. For example, while ADAS11, MMSE, and CDRSB show a large overlap in terms of value distribution (Supplementary Fig. S2), their respective trajectories in Figure 3 show a clear distinction in acceleration. In contrast, there are no notable group-wise differences in the trajectories for FDG-PET, despite the large difference in the biomarker distributions, We finally observe that the fast progressors show evidence of accelerated ventricular expansion, as well as pathological worsening of ADAS11 and MMSE. The confidence of DPMoSt for the specificity of these markers is close to the nominal significance value $\mathbb{P}(\xi_{b}^{2})=\mathbb{P}(1-\xi_{b}^{1})=\mathbb{P}(\text{split of biomarker b})=0.5$ .

It is interesting to observe that when applying DPMoSt only to brain’s volumetric features (ventricles, hippocampus, and entorhinal), we identify two clear progression subtypes, in which the fast progressors are associated with pathological deviations of the trajectories for the subcortical features (hippocampus and entorhinal), and a manifestly accelerated progression for the ventricles (Supplementary Fig. S1). While this analysis confirms the existence of distinct trajectories subtypes for the brain’s features alone, the larger variability induced by clinical scores, AV45 and FDG-PET, information describes in Figure 3 different progression subtypes better associated with the heterogeneity of the multimodal information.

##### 3.2.2.2 Comparison between subtypes progression profiles

**Fig. 4.**
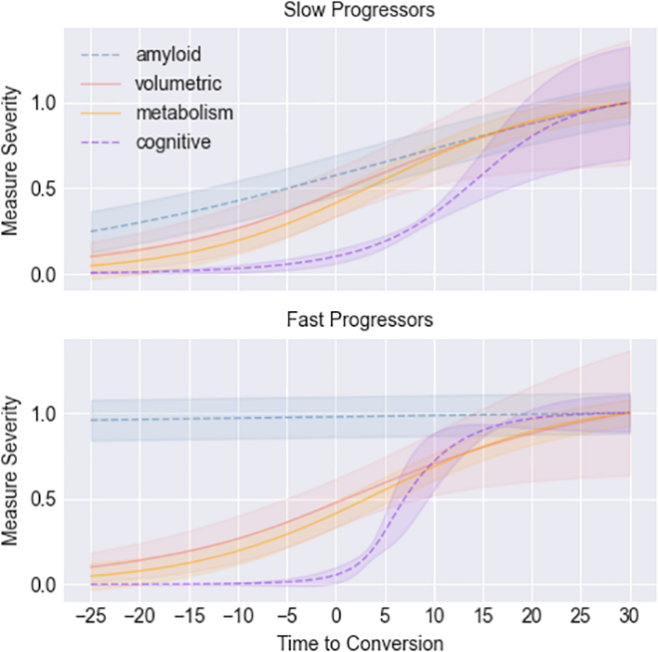
The figure illustrates the progression of the average biomarker values across the two subtypes identified by DPMoSt, subdivided by biomarker types: amyloid, volumetric, metabolic, and functional. Continuous lines represent biomarkers showing no difference between the slow and fast progressors, while dashed lines indicate those with distinct differences. We observe that the amyloid cascade hypothesis (Jack et al., 2010) holds true for both subtypes, with an accelerated progression in amyloid accumulation and functional decline in the fast progressors. The colored bands indicate the uncertainty in the average trajectories.

Figure 4 summarizes the estimated trajectories averaged across the macro-categories amyloid, volumetric, metabolism, and cognitive. Solid lines represent equal progression patterns between subtypes as estimated by DPMoSt, while dotted lines illustrate differences in the rate of degeneration between subtypes. The curves are generally in agreement with the amyloid cascade hypothesis (Jack et al., 2010), albeit with different progression rate between subtypes. In particular, amyloid levels are already in the plateau in the fast progressors population, while the cognitive markers deteriorate earlier and more rapidly.

##### 3.2.2.3 Genetic and CSF biomarkers differences between subtypes

A post hoc analysis (Table 4) shows that the fast progressors are characterized by significantly higher prevalence of the APOE4 allele (Cauchy combination test p<0.001 Bonferroni corrected for multiple comparisons). This subtype is also associated with elevated tau and phosphorylated tau (ptau) and reduced amyloid-beta (Aβ) levels in the CSF, indicating a more severe AD stage for these individuals (t-test p<0.001 Bonferroni corrected for multiple comparisons).

**Table 4.** Statistical significance between subtypes within each method is indicated in bold when p<0.001 , considering t-test or Cauchy-test when relevant, and correcting the *p*-value for multiple comparisons.

	DPMoSt		Leaspy		SuStaIn	
Biomarker	Slow Progr.	Fast Progr.	Slow Progr.	Fast Progr.	Slow Progr.	Fast Progr.
N subjects	225	227	430	22	307	145
Age (years)	72±6	74±7	73±7	71±9	73±7	75±8
Gender (F,M)	50%;50%	43%;57%	46%;54%	53%;47%	50%;50%	36%;64%
Education (years)	16.2±2.5	16.0±2.8	16.0±2.6	15.2±3.7	16.2±2.5	15.0±3.0
APOE ε4(0,1,2)	70%;27%;3%	45%;42%;13%	58%;34%;8%	60%;40%;0%	66%;30%;4%	40%;46%;14%
A β (pg/ml)	120±46	73±38	97±49	67±28	109±48	66±33
pTau (pg/ml)	23±11	33±16	28±14	35±17	27±14	32±15
Tau (pg/ml)	251±101	337±142	293±130	340±147	280±123	330±140
Between-subtype partition
Gender (F,M)	53%;47%	47%;53%	96%;97%	4%;3%	75%;62%	25%;38%
APOE ε4(0,1,2)	60%;38%;19%	40%;62%;81%	96%;95%;100%	4%;5%;0%	78%;57%;44%	22%;43%;56%

The table is divided into two panels: the panel above highlights the distribution within each subtype, while the panel below emphasizes the differences between the two discovered subtypes. While Leaspy fails to provide a meaningful subdivision, both DPMoSt and SuStaIn successfully distinguish between slow and fast progressors. Furthermore, when comparing the proportions of APOE4 carriers and non-carriers across the two subtypes, DPMoSt demonstrates a more pronounced prevalence of carriers in the fast progressor groups (bottom panel, last row).

The prevalence of the APOE4 allele among fast progressors becomes more evident when examining the differences between the two subtypes. Specifically, there is a higher prevalence of APOE4 alleles 1 and 2 in the fast progressors subtype, whereas the slow progressors subtype is characterized by a greater prevalence of the APOE4 allele 0.

Interestingly, despite the clear differences in biomarker progression, the analysis reveals no significant age difference between the subgroups, nor differences in education or gender distributions. This suggests that the acceleration of the fast progressors may be related to genetic predispositions, rather than demographic factors, further supporting the role of APOE4 in modulating the progression trajectory.

#### 3.2.3 Leaspy and SuStaIn results

##### 3.2.3.1 Subtypes inference

**Fig. 5.**
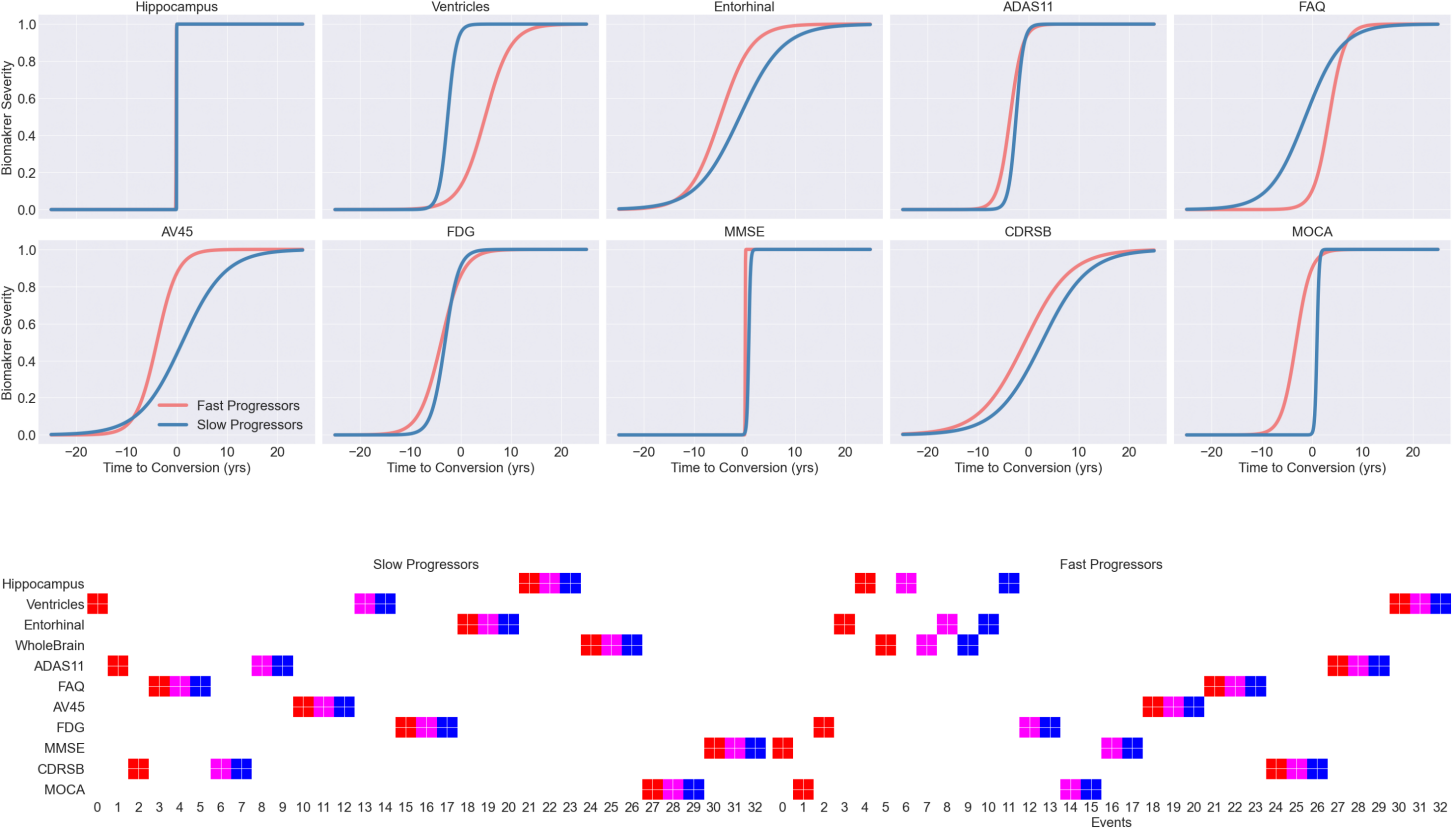
The figure illustrates the results of applying Leaspy (top row) and SuStaIn (bottom row) to the ADNI dataset. In the top row, the trajectories of the two identified subtypes are displayed, highlighted in blue and red. The bottom row presents the ordered events estimated by SuStaIn for the two subtypes as positional variance diagrams where each entry represents the probability that a particular scored event appears at a particular position along the progression pattern, with severity light shown in red, mild in magenta, and pathological in blue (Young et al., 2021). We observe that, while Leaspy fails to provide clinically meaningful results, SuStaIn successfully identifies ordered events with a clear distinction between volumetric decline and cognitive decline.

Figure 5 illustrates the results obtained by applying Leaspy (first panel) and SuStaIn (second panel) to the study population.

Leaspy identifies two distinct trajectory subtypes (blue and red curves) associated with 430 and 22 subjects, respectively. We observe notable issues arising with the Hippocampus biomarker and MMSE, where Leaspy repeatedly produces step-function trajectories across multiple runs. Furthermore, we note an unbalanced subdivision into subtypes; specifically, the two populations are skewed toward slow progressors, with only 22 subjects classified as fast progressors.

However, SuStaIn identifies two distinct trajectory subtypes associated with 145 and 307 subjects, respectively. The sequences of the events are significantly different with respect to the evolution of volumetric and clinical scores, whereby the fast progressors are associated with earlier progression onset than the slow progressors. SuStaIn clearly identifies two distinct subtypes characterized by late volumetric and cognitive decline (slow progressors) and early volumetric and cognitive decline (fast progressors). Interestingly, there appears to be a misalignment between the degeneration of volumetric measures and ventricle volumes in the fast progressors subtype.

##### 3.2.3.2 Genetic and CSF biomarkers differences between subtypes

Table 4 shows the results of the post hoc analysis for Leaspy and SuStaIn.

We observe that unlike DPMoSt, which successfully discerns subtypes based on genetic predisposition, Leaspy’s subtyping appears to be less effective. Specifically (i) the two subtypes show minimal differences in genetic and CSF biomarkers and (ii) slow progressors are significantly older than fast progressors.

In contrast, SuStaIn’s subtyping appears more informative than the one provided by Leaspy; indeed, the subdivisions within each subtype closely resemble those identified by DPMoSt, with significant differences between subtypes comparable with those observed with DPMoSt. However, when examining the differences between the two subtypes, SuStaIn primarily distinguishes them based on differences in the APOE4 allele 0, while evenly distributing the other two alleles. This indicates a reduced ability to differentiate between APOE4 carriers and non-carriers compared with DPMoSt.

### 3.3 Testing on external GMC cohort

In this section, we present the results of testing the SuStaIn and DPMoSt algorithms, which were previously trained on the ADNI dataset, using an external cohort. Testing with Leaspy was omitted due to its poor performance on the training data. Specifically, we focus on a dataset from the Geneva Memory Clinic at the Geneva University Hospitals (GMC). The dataset, which includes 171 subjects, 6 diagnosed with AD, 35 with MCI, and 130 classified as CN. Table 5 summarizes the baseline characteristics of this testing dataset. The study has been approved by the Commission cantonale d’éthique de la recherche—CCER de Genève; more details on the data collection processes are presented in Peretti et al. (2023). It is important to note that the information available in the testing dataset is more limited than the one available in the training one. Specifically, data for ADAS11, FAQ, AV45, FDG, and CDRSB are unavailable in this cohort.

**Table 5.** Baseline sociodemographic and clinical information for REDCAP test study cohort.

Group	CN	MCI	AD
N subjects	130	35	6
Age (years)	62 (8)	68 (7)	72 (6)
Gender (%F)	50	54	77
Education (1,2,3) %	1, 25, 74	0, 37, 73	0, 50, 50
Hippocampus (${\text{cm}}^{3}$)	5.1 (0.5)	4.2 (0.8)	3.7 (0.5)
Ventricles (${\text{cm}}^{3}$)	24.7 (17.2)	27.9 (18.5	24.7 (17.2)
Entorhinal (${\text{cm}}^{3}$)	2.8 (0.4)	2.1 (0.5)	1.9 (0.1)
MOCA	25 (2)	21 (4)	12 (9)
MMSE	28 (1)	26 (4)	16 (9)

CN: Cognitively Normal; MCI: Mild Cognitive Impairment; AD: Alzheimer’s disease.

To evaluate the algorithms’ performance, we assess their ability to accurately align subjects along the temporal trajectory (or events) relative to their diagnosis, and we examine their capacity to achieve significant discrimination in terms of a post hoc analysis of the prevalence of the APOE4 allele. Regarding the DPMoSt testing procedure, we considered fixed the approximated biomarker trajectories, noise levels, and split probabilities estimated during training; as a consequence, for new testing subjects, we optimized only the time-shift parameter and the probability of belonging to each subtype.

To assess whether the observed distribution of genotypes APOE4 0, 1, and 2 between subtypes is statistically significant, we conducted a two-sided permutation test. To this end, we first generated the null distribution of the histograms representing the prevalence of APOE4 (0, 1 and 2) in $10^{5}$ random subgroups having the same size of the estimated subtype. We then estimated the variability of the null distribution of APOE4 histograms by computing the Wasserstein distance between each sample and the reference APOE4 distribution in the full population. The resulting null distribution of Wasserstein distances allowed us to assess the two-sided *p*-value with respect to the distance associated with the APOE4 distribution identified by DPMoSt.

Regarding the application of SuStaIn, we adopted multivariate imputation to deal with missing features in the GMC dataset (Van Buuren & Groothuis-Oudshoorn, 2011). This approach was chosen to guarantee unbiasedness and improved variability estimates compared with the standard univariate imputation provided in the original implementation.

**Fig. 6.**
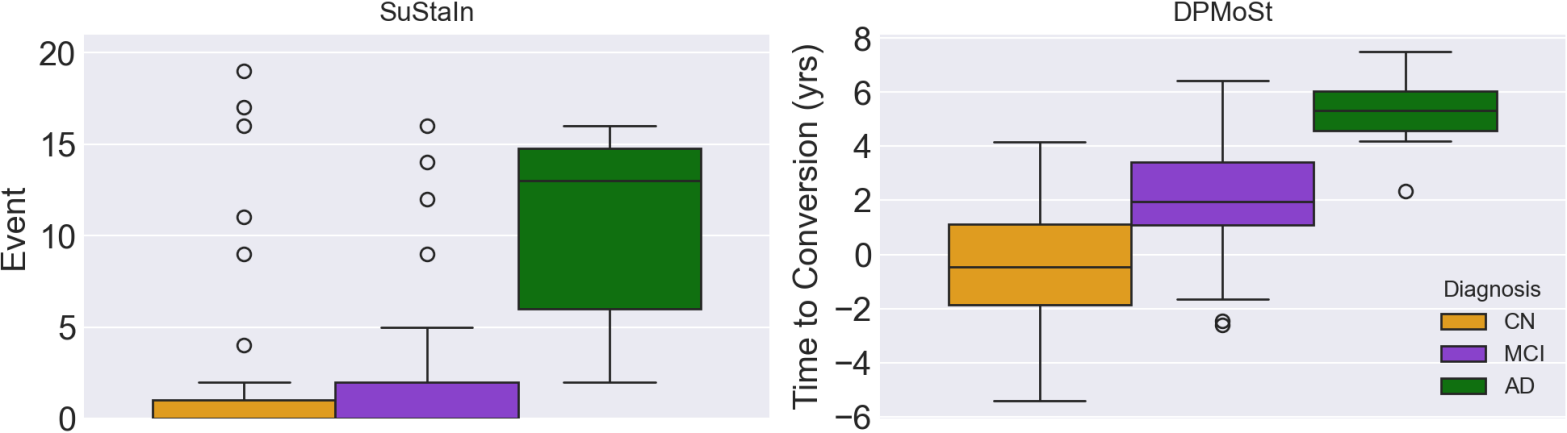
The figure shows the reparametrized time in the testing data, categorized by the subjects’ diagnoses for both methods SuStaIn and DPMoSt. We observe that the time re-parametrization by DPMoSt provides better separation of subjects’ time to conversion relative to their diagnosis compared with the one provided by SuStaIn.

DPMoSt classified 164 subjects into the Slow Progressors subtype and the remaining 7 subjects into the Fast Progressors subtype. The temporal alignment achieved through time-shift ordering (Fig. 6) effectively stratified patients based on their progression. Control subjects exhibited lower time shifts than MCI, while AD patients demonstrated the highest time shift. This progression pattern underscores the ability of the inferred trajectories to distinguish diagnostic groups during testing. Post hoc analysis confirmed that the inverse proportional relationship between Aβ and *p*-tau/tau observed in the training data was successfully preserved in the testing cohort. Moreover, the APOE4 allele distribution across the two subgroups demonstrated statistical significance, aligning with the findings of the training dataset (p<0.01 Bonferroni corrected for multiple comparisons).

However, SuStaIn assigned 148 subjects to the slow progressors subtype and the remaining 23 subjects to the fast progressors subtype. Analyzing the temporal alignment achieved through event ordering (Fig. 6, second subplot), we observed that SuStaIn was able to temporally separate AD subjects from MCI and CN in a meaningful way. However, there was no clear separation between MCI and control groups. Moreover the groups were not significantly different with respect to APOE4 distribution in the post hoc analysis (*p*-value >0.1 ).

## 4 Discussion

In this paper we presented DPMoSt, a new DPM method for automatic inference of subtypes in longitudinal datasets. DPMoSt addresses a critical aspect of DPM by allowing the estimation of biomarker specificity in subtype definition. We validated our method on synthetic and real datasets by comparing its performance with two established algorithms, Leaspy and SuStaIn.

Our analysis revealed that DPMoSt and SuStaIn perform similarly on the training data, both outperforming Leaspy in distinguishing between APOE4 carrier and non-carrier subtypes. In addition, DPMoSt offers high interpretability providing direct probabilities for a subject belonging to a specific subtype or for a biomarker exhibiting a split, capabilities that are lacking in current methods from the state-of-the-art.

The difference between DPMoSt and SuStaIn arises directly from our modeling assumptions, which set our approach apart from SuStaIn. While SuStaIn emphasizes clustering based on the magnitude of biomarker values to separate clinical groups, our method emphasizes differences in the progression speed or pattern over time. We acknowledge that this difference in modeling philosophy can lead to markedly different interpretations of the data and the resulting subtypes.

The ability to distinguish between informative and non-informative biomarkers is crucial for generalization purposes, as it can be used to prioritize biomarkers and discard non-informative ones in characterizing disease progression. In this sense, the assessment of the biomarker specificity provided by DPMoSt can be interpreted as a data-driven feature selection method for longitudinal data analysis. We observe that the primary difference between the two subpopulations of the ADNI cohort lies in the growth rate of the sigmoid trajectories, rather than in their midpoints. Nonetheless, we emphasize that the model is general and, in principle, capable of disentangling and accurately identifying subtypes that differ in midpoint, growth rate, or both—depending on the structure of the underlying data.

Moreover, identifying only relevant biomarkers facilitates the generalization of the method on external cohorts as shown in the application on the GMC dataset. In this case, we do not require the full set of biomarkers used during training, and we can make use of a limited set of measurements which corresponds to relevant features identified during training. Thanks to this ability, the subtype-specific biomarkers identified by DPMoSt in ADNI allowed to successfully generalize the results to the more challenging GMC memory clinic dataset, less populated and providing fewer biomarkers. These results highlight the robustness of DPMoSt as well as its potential for broader applicability in both clinical and research settings, offering a powerful tool for studying disease progression and subtype differentiation across diverse populations.

From a clinical point of view, the availability of an effective approach for subtyping individuals according to disease progression is essential to identify different patterns related to specific subtype characteristics. In particular, understanding how genetic predispositions and biomarker profiles influence disease progression can inform the development of more targeted and effective therapeutic strategies. Ultimately, the availability of a meaningful approach to subtyping is essential in clinical trials, as it ensures that cohorts are more homogeneous in terms of disease progression trajectories. This information is critical to help design consistent cohorts to maximize treatment efficacy by ensuring that individuals receive the interventions best suited to their specific disease subtype.

## Supplementary Material

Supplementary Material

## Data Availability

The simulated data and the code are available at https://gitlab.inria.fr/aviani/dpmost.
