# Supplementary material for: Disease Progression Modeling and Stratification for detecting sub-trajectories in the natural history of pathologies: Application to Alzheimer’s disease trajectory modeling
Source: Imaging Neurosci (Camb). 2025 Nov 6;3:IMAG.a.954. doi: 10.1162/IMAG.a.954 (PMC12592369; doi:10.1162/IMAG.a.954)
Supplement: Supplementary Material [file IMAG.a.954_supp.pdf]

Supplementary Material

Disease Progression Modeling and Stratification for Detecting Sub-trajectories in the Natural History of Pathologies: Application to Alzheimer's Disease Trajectory Modeling

2025-09-28

A EM for the optimization

In this Section we give the mathematical details for performing the EM-steps needed for the evaluation of the MAP of the posterior distribution of the model (6) in the case in which only two sub-trajectories are considered:

$$p(\boldsymbol{\theta}, \boldsymbol{\sigma}, \boldsymbol{\xi}, \boldsymbol{\pi}, \boldsymbol{\tau} \mid \boldsymbol{x}) \; . \tag{1}$$

We decided to exploit the two level mixture model structure to implement a multiple step approach. Indeed we iteratively perform the optimization of the parameters $\boldsymbol{\theta}$ via Stochastic Gradient Descent (SGD) and a classical EM step for the parameters $\boldsymbol{\xi}$ and $\boldsymbol{\pi}$.

For consistency with the main text, we denote the total number of subjects as J and the total number of biomarkers as B.

A.1 EM for subtype detection.

For what concerns the EM-step for the parameter $\boldsymbol{\xi}$, we follow the same reasoning used for deriving the classical EM-step, i.e. we first derive the posterior distribution for the split.

Let us introduce a new auxiliary random variable $z_b \in \{0, 1\}$ such that the event $z_b = 0$ corresponds to the event "there is a split" and the event $z_b = 1$ corresponds to the event 'there is no split'. As a

consequence, its density is given by:

$$p(z_b) = \begin{cases} \xi_b^1 & z_b = 1 \\ 1 - \xi_b^1 & z_b = 0 \end{cases} \tag{2}$$

Therefore we can describe the posterior distribution for the auxiliary variable given the data in terms of other quantities:

$$\begin{aligned} \gamma_j(\xi_b^1) = p(z_b = 1 \mid \boldsymbol{x}_j^b) &= \frac{p(\boldsymbol{x}_j^b \mid z_b = 1) p(z_b = 1)}{p(\boldsymbol{x}_j^b)} \\ &= \frac{p(\boldsymbol{x}_j^b \mid \bar{\boldsymbol{\theta}}_b^{(1,1)}) \xi_b^1}{p(\boldsymbol{x}_j^b)}; \end{aligned} \tag{3}$$

$$\begin{aligned} 1 - \gamma_j(\xi_b^1) = p(z_b = 0 \mid \boldsymbol{x}_j^b) &= \frac{p(\boldsymbol{x}_j^b \mid z_b = 0) p(z_b = 0)}{p(\boldsymbol{x}_j^b)} \\ &= \frac{p(\boldsymbol{x}_j^b \mid \bar{\boldsymbol{\theta}}_b^{(2,1)}, \bar{\boldsymbol{\theta}}_b^{(2,2)})(1 - \xi_b^1)}{p(\boldsymbol{x}_j^b)}\ , \end{aligned} \tag{4}$$

where the conditioning on noise standard deviation is omitted for simplicity of notation.

For performing the EM-step, we can consider to evaluate the gradient of the posterior distribution w.r.t. the parameter of interest and setting it equal to zero:

$$\begin{aligned} \partial_{\xi_b} \left(\ln(p(\boldsymbol{\theta}, \boldsymbol{\sigma}, \boldsymbol{\xi}, \boldsymbol{\pi}, \boldsymbol{\tau} \mid \boldsymbol{x})) \right) &= \partial_{\xi_b^1} \left(\ln(p(\boldsymbol{x} \mid \boldsymbol{\theta}, \boldsymbol{\sigma}, \boldsymbol{\xi}, \boldsymbol{\pi}, \boldsymbol{\tau})) + \beta_\xi \xi_b^1 \right) \\ &= \sum_j \left(\frac{\partial_{\xi_b^1} p(\boldsymbol{x}_j^b \mid \boldsymbol{\theta}, \sigma_b, \xi_b, \pi_j, \tau_j)}{p(\boldsymbol{x}_j^b \mid \boldsymbol{\theta}, \sigma_b, \xi_b^1, \pi_j, \tau_j)} \right) + \beta_\xi \\ &= \sum_j \left(\frac{p(\boldsymbol{x}_j^b \mid \bar{\boldsymbol{\theta}}_b^{(1,1)}, \sigma_b, \xi_b^1, \pi_j, \tau_j) - p(\boldsymbol{x}_j^b \mid \bar{\boldsymbol{\theta}}_b^{(2,1:2)}, \sigma_b, \xi_b, \pi_j, \tau_j)}{p(\boldsymbol{x}_j^b)} \right) + \beta_\xi \\ &= \sum_j \left(\gamma_j(\xi_b^1) - \xi_b^1 \right) + \beta_\xi (1 - \xi_b^1) \xi_b^1 \\ &= \sum_j \gamma_j(\xi_b^1) + (-J + \beta_\xi (1 - \xi_b^1)) \xi_b^1\ . \end{aligned}$$

Therefore, if we want to maximize the value for ξ_b we can equalize to zero the loss function derivative,

obtaining an iterative way to update the parameter:

$$\xi_b^{1,new} = \frac{\sum_j \gamma_j(\xi_b^{1,old})}{J + (\xi_b^{1,old} - 1)\beta_\xi} . \tag{5}$$

We observe that, due to the fact that ξ_b is a mixture coefficient, it has to be a value in range between zero and one; therefore not all values for the prior parameter β_ξ can be considered. In the next Section we are giving a sufficient condition to ensure ξ_b to be in an appropriate range.

Bounds for prior parameter. In this Section we derive a sufficient condition on the prior parameter $\beta_x i$ to allow ξ to be a proper mixture coefficient for our model, i.e. between zero and one.

This Lemma is useful for the demonstration of the following result.

Lemma 1. *Let f and g be non negative functions in D; then for all $x_1, \ldots, x_n \in D$, the following inequality holds:*

$$\frac{\sum_{i=1}^n f(x_i)}{\sum_{i=1}^n g(x_i)} < \sum_{i=1}^n \frac{f(x_i)}{g(x_i)}.$$

Proof. The proof is straightforward, indeed, being g a non negative function, for a given $x_i \in D$ we have that:

$$\frac{f(x_i)}{\sum_{j=1}^n g(x_j)} \leq \frac{f(x_i)}{g(x_i)} .$$

By summing both side of the inequality across all possible $i = 1, \ldots, n$, we obtain the thesis:

$$\frac{\sum_{i=1}^n f(x_i)}{\sum_{j=1}^n g(x_j)} = \sum_{i=1}^n \frac{f(x_i)}{\sum_{j=1}^n g(x_j)} \leq \sum_{i=1}^n \frac{f(x_i)}{g(x_i)} .$$

□

Lemma 2. *Given $p(\boldsymbol{\theta}, \boldsymbol{\sigma}, \boldsymbol{\xi}, \boldsymbol{\pi}, \boldsymbol{\tau} \mid \boldsymbol{x})$, if the likelihood with two sub-trajectories is in average better than the one with one trajectory, then, for each biomarker:*

$$\frac{\sum_j p(\boldsymbol{x}_j^b \mid \bar{\boldsymbol{\theta}}_b^{(2,1:2)}) - \Big(p(\boldsymbol{x}_j^b \mid \bar{\boldsymbol{\theta}}_b^{(1,1)}) - p(x_j^b \mid \bar{\boldsymbol{\theta}}_b^{(2,1:2)})\Big)\, \xi_b^{1,old}}{\sum_j p(\boldsymbol{x}_j^b \mid \bar{\boldsymbol{\theta}}_b^{(2,1:2)}) + \Big(p(\boldsymbol{x}_j^b \mid \bar{\boldsymbol{\theta}}_b^{(1,1)}) - p(\boldsymbol{x}_j^b \mid \bar{\boldsymbol{\theta}}_b^{(2,1:2)})\Big)\, \xi_b^{1,old}} > 1 .$$

Proof.

$$\begin{aligned}
&\sum_j p(x_j^b \mid \bar{\boldsymbol{\theta}}_b^{(2,1:2)}) > \sum_j p(x_j^b \mid \bar{\boldsymbol{\theta}}_b^{(1,1)}) \\
&\Leftrightarrow \sum_j -2p(x_j^b \mid \bar{\boldsymbol{\theta}}_b^{(1,1)}) + 2p(x_j^b \mid \bar{\boldsymbol{\theta}}_b^{(2,1:2)}) > 0 \\
&\Leftrightarrow \sum_j -p(x_j^b \mid \bar{\boldsymbol{\theta}}_b^{(1,1)}) + p(x_j^b \mid \bar{\boldsymbol{\theta}}_b^{(2,1:2)}) - p(x_j^b \mid \bar{\boldsymbol{\theta}}_b^{(1,1)}) + p(x_j^b \mid \bar{\boldsymbol{\theta}}_b^{(2,1:2)}) > 0 \\
&\Leftrightarrow \sum_j p(x_j^b \mid \bar{\boldsymbol{\theta}}_b^{(2,1:2)}) - \Big(p(x_j^b \mid \bar{\boldsymbol{\theta}}_b^{(1,1)}) - p(x_j^b \mid \bar{\boldsymbol{\theta}}_b^{(2,1:2)})\Big)\, \xi_b^1 - p(x_j^b \mid \bar{\boldsymbol{\theta}}_b^{(2,1:2)}) - \\
&\Big(p(x_j^b \mid \bar{\boldsymbol{\theta}}_b^{(1,1)}) - p(x_j^b \mid \bar{\boldsymbol{\theta}}_b^{(2,1:2)})\Big)\, \xi_b^1 > 0 \ ,
\end{aligned}$$

from which we obtain the thesis. □

Theorem 1. *Given $p(\boldsymbol{\theta}, \boldsymbol{\sigma}, \boldsymbol{\xi}, \boldsymbol{\pi}, \boldsymbol{\tau} \mid \boldsymbol{x})$, if the likelihood with two sub-trajectories is in average better than the one with one trajectory, $\xi^{1,(\ell-1)} \in [0,1]$, $\xi^{1,(\ell)}$ is given by (5), and $\beta_\xi \in [0, J]$, then $\xi_b^{1,new} \in [0,1]$.*

Proof. We start proving that $0 \leq \beta_\xi \leq J$ ensures $\xi_b^{1,new} \geq 0$. We observe that by equation (5) we have that

$$\begin{aligned}
\xi_b^{1,new} \geq 0 &\Leftrightarrow \frac{\sum_j \gamma_j(\xi_b^{1,old})}{J + (\xi_b^{1,old} - 1)\beta_\xi} \geq 0 \\
&\Leftrightarrow J + (\xi_b^{1,old} - 1)\beta_\xi \geq 0 \\
&\Leftrightarrow \beta_\xi \leq \frac{J}{1 - \xi_b^{1,old}} \ ;
\end{aligned}$$

Therefore, being $N \leq N/(1 - \xi_b^{1,old})$, we obtain the sufficient condition $\beta_\xi \leq N$.

We now prove that $0 \leq \beta \leq N$ ensures $\xi_b^{1,new} \leq 1$.

We observe again that by equation (5) we have that

$$
\begin{aligned}
\xi_b^{1,new} \leq 1 \Leftrightarrow \frac{\sum_j \gamma_b^n}{N + (\xi_b^{1,old} - 1)\beta_\xi} \leq 1 \\
\Leftrightarrow \beta_\xi \leq \frac{1}{1 - \xi_b^{1,old}} \sum_j (1 - \gamma_b^n) \\
\Leftrightarrow \beta_\xi \leq \frac{1}{1 - \xi_b^{1,old}} \sum_j \frac{p(x_j^b \mid \bar{\boldsymbol{\theta}}_b^{(2,1:2)})(1 - \xi_b^{1,old})}{p(x_j^b)} \\
\Leftrightarrow \beta_\xi \leq \sum_j \frac{p(x_j^b \mid \bar{\boldsymbol{\theta}}_b^{(2,1:2)})}{p(x_j^b)} \,.
\end{aligned}
$$

We observe that the upper bound can be written as:

$$
\begin{aligned}
\sum_j \frac{p(x_j^b \mid \bar{\boldsymbol{\theta}}_b^{(2,1:2)})}{p(x_j^b)} &= \sum_j \frac{p(x_j^b \mid \bar{\boldsymbol{\theta}}_b^{(2,1:2)})}{p(x_j^b \mid \bar{\boldsymbol{\theta}}_b^{(2,1:2)})(1 - \xi_b^{1,old}) + p(x_j^b \mid \bar{\boldsymbol{\theta}}_b^{(1,1)})\xi_b^{1,old}} \\
&= \sum_j \frac{p(x_j^b \mid \bar{\boldsymbol{\theta}}_b^{(2,1:2)})}{p(x_j^b \mid \bar{\boldsymbol{\theta}}_b^{(2,1:2)}) + \left(-p(x_j^b \mid \bar{\boldsymbol{\theta}}_b^{(2,1:2)}) + p(x_j^b \mid \bar{\boldsymbol{\theta}}_b^{(1,1)})\right) \xi_b^{1,old}} \\
&= \sum_j \frac{p(x_j^b \mid \bar{\boldsymbol{\theta}}_b^{(2,1:2)}) \pm \left(p(x_j^b \mid \bar{\boldsymbol{\theta}}_b^{(1,1)}) - p(x_j^b \mid \bar{\boldsymbol{\theta}}_b^{(2,1:2)})\right) \xi_b}{p(x_j^b \mid \bar{\boldsymbol{\theta}}_b^{(2,1:2)}) + \left(p(x_j^b \mid \bar{\boldsymbol{\theta}}_b^{(1,1)}) - p(x_j^b \mid \bar{\boldsymbol{\theta}}_b^{(2,1:2)})\right) \xi_b^{1,old}} \\
&\geq \frac{\sum_j p(x_j^b \mid \bar{\boldsymbol{\theta}}_b^{(2,1:2)}) \pm \left(p(x_j^b \mid \bar{\boldsymbol{\theta}}_b^{(1,1)}) - p(x_j^b \mid \bar{\boldsymbol{\theta}}_b^{(2,1:2)})\right) \xi_b^{1,old}}{\sum_j p(x_j^b \mid \bar{\boldsymbol{\theta}}_b^{(2,1:2)}) + \left(p(x_j^b \mid \bar{\boldsymbol{\theta}}_b^{(1,1)}) - p(x_j^b \mid \bar{\boldsymbol{\theta}}_b^{(2,1:2)})\right) \xi_b^{1,old}} \\
&= J + \frac{\sum_j p(x_j^b \mid \bar{\boldsymbol{\theta}}_b^{(2,1:2)}) - \left(p(x_j^b \mid \bar{\boldsymbol{\theta}}_b^{(1,1)}) - p(x_j^b \mid \bar{\boldsymbol{\theta}}_b^{(2,1:2)})\right) \xi_b^{1,old}}{\sum_j p(x_j^b \mid \bar{\boldsymbol{\theta}}_b^{(2,1:2)}) + \left(p(x_j^b \mid \bar{\boldsymbol{\theta}}_b^{(1,1)}) - p(x_j^b \mid \bar{\boldsymbol{\theta}}_b^{(2,1:2)})\right) \xi_b^{1,old}} \\
&> J \,.
\end{aligned}
$$

where the inequalities come from the previous Lemma 1 and Lemma 2.

This means that the bound for β_ξ holds if the model with two Sigmoids is in general better than the one with one Sigmoid. This requirement is reasonable.

Therefore, if the ratio of sums on the right hand side is positive, it is true that $\beta_\xi < J$ is a good bound. □

A.2 EM for subtype partition

For what concerns the parameters $\boldsymbol{\pi}$, the reasoning is similar, with the simplicity given by the fact that it is a common EM algorithm. For convenience of notation, in the following, we will indicate $\tilde{\pi}_j = \pi_j^{(2,1)}$.

Let us introduce a new variable $\nu^n \in \{0, 1\}$ such that

$$p(\nu_j) = \begin{cases} \tilde{\pi}_j & \nu_j = 1 \\ 1 - \tilde{\pi}_j & \nu_j = 0 \end{cases} \tag{6}$$

Therefore we can describe the posterior distribution for the auxiliary variable in terms of other quantities:

Following the same reasoning

$$\begin{aligned} \chi_b(\tilde{\pi}_j) = p(\nu_j = 1 \mid \boldsymbol{x}_j^b) &= \frac{p(\boldsymbol{x}_j^b \mid \nu_j = 1) p(\nu_j = 1)}{p(\boldsymbol{x}_j^b)} \\ &= \frac{p(\boldsymbol{x}_j^b \mid \bar{\boldsymbol{\theta}}_b^{(2,2)}) \tilde{\pi}_j}{p(\boldsymbol{x}_j^b)} ; \end{aligned} \tag{7}$$

$$\begin{aligned} 1 - \chi_b(\tilde{\pi}_j) = p(\nu_j = 0 \mid \boldsymbol{x}_j^b) &= \frac{p(\boldsymbol{x}_j^b \mid \nu_j = 0) p(\nu_j = 0)}{p(\boldsymbol{x}_j^b)} \\ &= \frac{p(\boldsymbol{x}_j^b \mid \bar{\boldsymbol{\theta}}_b^{(2,2)}) (1 - \tilde{\pi}_j)}{p(\boldsymbol{x}_j^b)} . \end{aligned} \tag{8}$$

For performing the EM-step, we can consider to evaluate the gradient of the loss function and setting it equal to zero:

$$\begin{aligned} \partial_{\tilde{\pi}_j} \left(\ln(p(\boldsymbol{\theta}, \boldsymbol{\sigma}, \boldsymbol{\xi}, \boldsymbol{\pi}, \tau \mid \boldsymbol{x})) \right) &= \sum_b \frac{\partial_{\tilde{\pi}_j} p(\boldsymbol{x}_j^b \mid \boldsymbol{\theta}, \sigma_b, \xi_b, \tilde{\pi}_j, \tau_j)}{p(\boldsymbol{x}_j^b \mid \theta_b, \sigma_b, \xi_b, \tilde{\pi}_j, \tau_j)} \\ &= \sum_b \chi_b(\tilde{\pi}_j) - J \tilde{\pi}_j . \end{aligned} \tag{9}$$

Therefore, if we want to maximize the value for π^n we can equalize to zero the loss function derivative,

obtaining an iterative way to update the parameter:

$$\tilde{\pi}_j^{new} = \frac{1}{J} \sum_b \chi_b(\tilde{\pi}_j^{old}) \, . \tag{10}$$

B Supplementary Figures

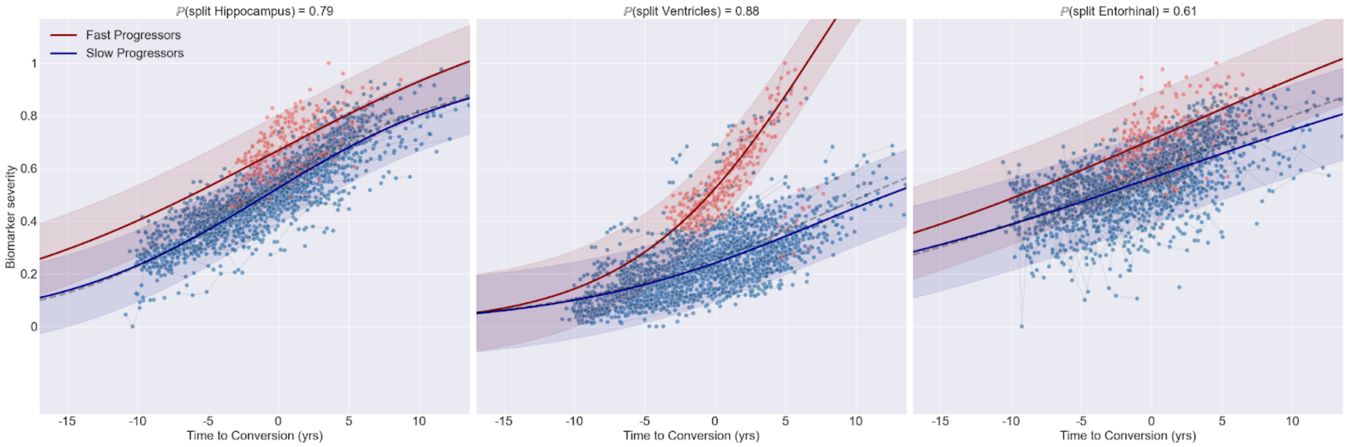

Figure S1: DpMost trajectory clustering applied to the brain's volumetric features only (hippocampus, ventricles and entorhinal).

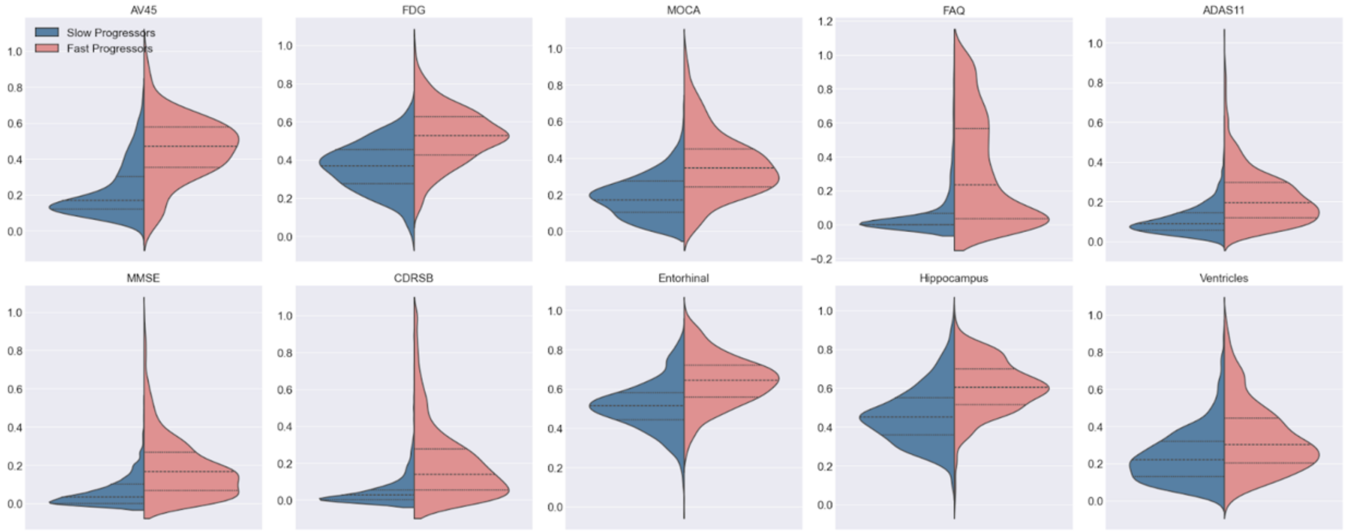

Figure S2: Distribution of biomarkers between subtypes on the ADNI dataset, ordered by statistical difference (top-left to bottom-right).
